# H3 relaxin protects against calcium oxalate crystal‐induced renal inflammatory pyroptosis

**DOI:** 10.1111/cpr.12902

**Published:** 2020-09-18

**Authors:** Jiannan Liu, Kelaier Yang, Yinshan Jin, Yadong Liu, Yaodong Chen, Xiaohui Zhang, Shiliang Yu, Erlin Song, Song Chen, Jingbo Zhang, Guanhua Jing, Ruihua An

**Affiliations:** ^1^ Department of Urology The First Affiliated Hospital of Harbin Medical University Heilongjiang China; ^2^ Department of Endocrinology The First Affiliated Hospital of Harbin Medical University Heilongjiang China; ^3^ Department of Urology Ningbo First Hospital Zhejiang China; ^4^ Department of Ultrasonic Imaging The First Affiliated Hospital of Shanxi Medical University Shanxi China; ^5^ Department of Cardiology The First Affiliated Hospital of Harbin Medical University Heilongjiang China

**Keywords:** cytokines, H3 relaxin, inflammation, kidney, oxidative injury, pyroptosis

## Abstract

**Objectives:**

Calcium oxalate (CaOx) crystals can activate inflammatory cytokines by triggering inflammasomes, which cause damage to the adhered epithelium, a dysfunctional microenvironment and even renal failure. However, a comprehensive and in‐depth understanding of the mechanisms underlying the effects of these crystals on damage and cytokine function in renal tubular epithelial cells (TECs) remains limited and to be explored.

**Materials and Methods:**

We detected the pyroptosis of TECs induced after exposure to CaOx crystals and demonstrated the significance of cytokine activation in the subsequent inflammatory processes through a proteomic study. We then conducted animal and cell experiments to verify relevant mechanisms through morphological, protein, histological and biochemical approaches. Human serum samples were further tested to help explain the pathophysiological mechanism of H3 relaxin.

**Results:**

We verified that crystal‐induced extracellular adenosine triphosphate (ATP) upregulation via the membrane purinergic 2X_7_ receptor (P2X_7_R) promotes ROS generation and thereby activates NLRP3 inflammasome‐mediated interleukin‐1β/18 maturation and gasdermin D cleavage. Human recombinant relaxin‐3 (H3 relaxin) can act on the transmembrane receptor RXFP1 to produce cAMP and subsequently improves crystal‐derived damage via ATP consumption. Additionally, endogenous relaxin‐3 was found to be elevated in patients with renal calculus and can thus serve as a biomarker.

**Conclusions:**

Our results provide previously unidentified mechanistic insights into CaOx crystal‐induced inflammatory pyroptotic damage and H3 relaxin‐mediated anti‐inflammatory protection and thus suggest a series of potential therapeutic targets and methods for but not limited to nephrocalcinosis.

## Graphical abstract

Calcium oxalate crystals deposition causes renal inflammatory pyroptosis and may be protected by H3 relaxin via action on receptor RXFP‐1.

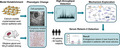

## 1 INTRODUCTION

Crystal‐related renal injury is usually due to congenital or acquired metabolic disturbances, which can be induced and magnified by various factors, such as local anatomical or humoral abnormalities.^1^, ^2^ Calcium oxalate (CaOx) crystal formation secondary to hyperoxaluria accounts for the vast majority of cases of calculous disease.^3^ From nephrocalcinosis to symptomatic stone incarceration and even renal failure, the adhesion of crystals, ranging from microcosmic to macroscopic, involves different mechanisms and results in different degrees of damage to the kidney, including inflammation, oxidative stress and fibrosis,^4^ and the renal tubular epithelium to which the crystals adhere is the primary and direct site of these pathological changes.^5^ The previous therapies for renal function impairment induced by crystalline nephropathies principally focused on the removal of anatomical factors and the reduction in serum or urine oxalate.^6^ The available research aimed at blunting the damage induced by CaOx crystal deposition and preserving renal function remains limited, and an in‐depth exploration has not been performed.

The inflammatory response is involved in the complete process of crystalline nephropathies from the onset to the end stage, and during this process, the activation of the innate immune system by the inflammasome plays a crucial role.^7^ The nucleotide‐binding domain and leucine‐rich repeat‐containing (NLR) family pyrin domain‐containing 3 (NLRP3) inflammasome, which is a protein complex composed of a sensor, an adaptor and an effector (NLRP3, apoptosis‐associated speck‐like protein containing a CARD [ASC] and pro‐caspase‐1, respectively), participate in CaOx crystal‐induced renal injury, and the regulation of its upstream and downstream axes has been assessed in different models.^8^, ^9^ Many factors are thought to trigger or be involved in two functionally distinct steps, namely the priming and activation of the NLRP3 inflammasome, including K^+^ efflux, particle uptake, lysosomal lysis, and excess adenosine triphosphate (ATP) or reactive oxygen species (ROS) production.^10^ However, the theories related to these factors were developed relatively independently and are not consistent. We have proven that ATP‐coupled purinergic 2X_7_ receptor (P2X_7_R) positively regulates the NLRP3 inflammasome in the presence of ROS, but the mechanism and targets of CaOx crystals associated with the activation of P2X_7_R and the triggering of the NLRP3 axis remain unclear.^11^, ^12^ Based on our knowledge of the canonical inflammasome pathway, cleaved caspase‐1 converts pro‐IL‐1β and pro‐IL‐18 to their mature forms, which are secreted extracellularly and then regulate inflammatory and immune responses.^13^ Recent studies showed that gasdermin D (GSDMD) is also cleaved by caspase‐1 to form an amino‐terminal fragment, which oligomerizes and generates pores on the cell membrane to induce pyroptosis, and these findings provide a new direction for research on crystalline nephropathy.^14^

Pyroptosis is a newly identified type of programmed cell death that is characterized by nuclear pyknosis, cell swelling and membrane rupture and exhibits morphological, biological and regulatory specificity.^15^ From an immunological perspective, classical pyroptosis essentially belongs to the inflammation process due to its dependence on the NLRP3‐caspase‐1 axis and the release of numerous inflammatory mediators.^14^, ^16^ The role of pyroptosis has gradually been demonstrated in various fields, including cancers, infectious diseases, chronic kidney diseases and crystalline nephropathy.^17^, ^18^, ^19^, ^20^ Whether CaOx crystal deposition results in pyroptotic damage in the kidney has not yet been explored, and this process might be a potential therapeutic target if both the validity of this hypothesis is confirmed and the process can be suppressed through the inflammatory axis.

We aimed to identify a mediator with anti‐inflammatory and antioxidative functions, few side effects and good tolerance in humans for the alleviation of inflammatory injury. The emerging polypeptide hormone relaxin, which is endogenously produced by many glands, such as the pituitary, ovary, prostate and other gonads, meets these standards.^21^ Relaxin family peptide receptors are widely distributed in the heart, brain, kidney and reproductive system and might trigger anti‐inflammatory, antioxidative, antiapoptotic and antifibrotic pathways after ligand binding.^22^, ^23^ The members of the relaxin family can be divided into subtypes, among which relaxin‐2 has been extensively studied, and its protective effects against heart failure have been clinically validated.^24^ Relaxin‐3 has also received some interest, and we confirmed the anti‐inflammatory and antioxidative effects of synthetic relaxin‐3 (H3 relaxin) in diabetic cardiomyopathy,^11^, ^25^ but its role in renal inflammation has not been explored. Notably, clinical studies have reported increased endogenous serum relaxin levels in some patients with chronic diseases, which suggests its involvement in self‐protective mechanisms and its potential as a biomarker of pathological severity and prognosis.^26^, ^27^ We therefore hypothesized that the serum relaxin‐3 level differs in patients with CaOx stones.

This investigation constitutes the first exploration of the existence of inflammatory pyroptosis in CaOx crystal‐related renal injury, and this exploration includes various analyses, ranging from phenotypic to mechanistic studies. We tested the hypothesis that this particular damage is strongly associated with the NLRP3‐caspase‐1 axis, which might be regulated by ATP, P2X_7_R, K^+^ and ROS, and then delineated the complete sequence of these processes. We also explored the protective mechanism of H3 relaxin in this nephrogenic inflammation process and clinically examined the associated endocrine changes. This study aimed to elucidate the inflammatory pyroptotic pathway and provide a series of therapeutic strategies for this type of injury.

## 2 MATERIALS AND METHODS

### 2.1 Cell culture studies

Human proximal tubule HK2 cells, which serve as tubular epithelial cells (TECs), were originally supplied by the American Type Culture Collection. Primary cells were maintained in Dulbecco’s modified Eagle’s medium (DMEM) (Gibco, Invitrogen) plus 10% FBS and 1% penicillin‐streptomycin and cultured in an incubator at 37°C with 5% CO_2_. The cell experiments were divided into steps according to experimental design. The cells were pretreated with 20 ng/mL H3 relaxin (035‐36A; Phoenix Pharmaceuticals) for 2 hours, 1 μmol/L H_2_O_2_ for 8 hours, 10 mmol/L N‐Acetyl‐L‐cysteine (NAC) for 12 hours, 10 mmol/L BzATP for 24 hours, 10 mmol/L A438079 for 24 hours, 1 μmol/L MCC950 for 24 hours, 20 μmol/L MitoTEMPO for 12 hours, 1 U/mL apyrase for 12 hours or 0.5 mmol/L 3‐isobutyl‐1‐methylxanthine (IBMX) for 24 hours (all purchased from Sigma‐Aldrich) and then stimulated with 0.5 mmol/l CaOx crystals for 24 hours as described previously.^11^, ^28^ Cell‐free supernatants or centrifuged cells were collected for biochemical analyses.

### 2.2 Preparation of plain CaOx crystals

Synthetic plain CaOx crystals were prepared as described previously.^29^ CaCl_2_·2H_2_O (10 mmol/L; Sigma‐Aldrich) and Na_2_C_2_O_4_ (10 mmol/L; Sigma‐Aldrich) were dissolved in deionized water and then mixed to a concentration of 5 mmol/L.^30^ The mixture was adjusted to pH 7.4 by HCl and NaOH, allows to settle overnight at room temperature and then centrifuged at 2000 *g* for 5 minutes. The CaOx crystals were washed with methanol, air‐dried and sterilized under UV light prior to use.

### 2.3 Animal studies

Male Sprague‐Dawley rats (150‐200 g, 5‐6 weeks old) were supplied by the Animal Research Center of Harbin Medical University and housed in a suitable environment with unlimited access to food and water. The study scheme followed the relevant protocol (Committee on the Use of Live Animals of Harbin Medical University, Regulation of US National Institutes of Health). An oxalate nephropathy model was induced by the addition of 0.8% ethylene glycol (EG) (Sigma‐Aldrich) and 0.8% NH_4_Cl (Sigma‐Aldrich) to drinking water as described previously.^31^ Sixty rats were randomly divided into four groups: the control group, the EG group, and the low‐ and high‐dose H3 relaxin groups. The low‐ and high‐dose H3 relaxin groups were fed as the EG group and subcutaneously injected with synthetic H3 relaxin at a dose of 0.2 and 2 μg/kg/d, respectively, as described previously.^32^ The kidneys were harvested, and plasma was obtained after 7 and 14 days of treatment. These samples were fixed or stored at −80°C for subsequent histological and biochemical analyses.

### 2.4 Biochemical assay

An enzyme‐linked immunosorbent assay (ELISA) Kit was used to measure the concentration of cAMP (H164; Nanjing Jiancheng Bioengineering Institute). The intracellular ATP, extracellular ATP (eATP) and ATP levels in tissue extracts were detected using a colorimetric ATP assay kit (Ab83355; Abcam). Serum creatinine (Scr) and blood urea nitrogen (BUN) were measured using appropriate kits (Ab65340 and Ab83362; Abcam).

### 2.5 Western blot analysis

Cells or homogenized kidney tissues were harvested in radioimmunoprecipitation assay buffer (Beyotime) and fully lysed on ice. After centrifugation (12 000 rpm, 10 minutes), the supernatants were collected, and the protein concentration was determined. Equal amounts of proteins were separated by SDS‐PAGE and then transferred onto poly(vinylidene fluoride) (PVDF) membranes. The membranes were blocked with 5% nonfat milk/Tris buffered saline with Tween (TBST) and successively incubated with the corresponding primary and secondary antibodies. The protein signals were then assessed using enhanced chemiluminescence (Thermo Fisher Scientific). The following primary antibodies were used: ASC (sc‐514414), PKA (sc‐390548), P2X_7_R (sc‐514962) and RXFP‐1 (sc‐293228) from Santa Cruz Biotech; cleaved caspase‐1 (4199/89332) and pro‐caspase‐1 (3866) from Cell Signaling Technology; IL‐1β (WL00891) and NLRP3 (WL02635) from Wanleibio (Wanleibio); cleaved GSDMD ([ab215203; Abcam], [sc‐393656; Santa Cruz Biotech]); IL‐18 (10663‐1‐AP; ProteinTech); and glyceraldehyde‐3‐phosphate dehydrogenase (GAPDH) (AC002; ABclonal). Peroxidase‐conjugated secondary antibodies (ZSGB‐BIO) were used.

### 2.6 Immunofluorescence

Cells seeded on 35‐mm glass‐bottom culture dishes were fixed with 4% paraformaldehyde and then incubated with blocking solution (goat serum containing 0.1% Triton X‐100). Subsequently, the cells were incubated with the appropriate primary antibodies (cleaved caspase‐1 [WL02996a; Wanleibio], GSDMD [sc‐81868; Santa Cruz Biotech]) and then with fluorescein isothiocyanate (FITC)‐conjugated secondary antibodies (ProteinTech). The cell nuclei were counterstained with 4',6‐diamidino‐2‐phenylindole (DAPI) prior to observation under a fluorescence microscope (BX53; Olympus).

### 2.7 Histology and immunohistochemistry staining

Rat kidneys were routinely processed for formaldehyde fixation, embedded in paraffin and positioned to obtain complete cross sections of the kidneys at the level of the renal papilla. Sections were first cut to a thickness of 4 μm and stained with haematoxylin and eosin (HE) for routine histological examination. Von Kossa staining was performed to visualize the crystals. Subsequently, the samples were sectioned for immunostaining with specific antibodies (cleaved caspase‐1 [WL02996a; Wanleibio], GSDMD [ab219800; Abcam]) against the target proteins. The procedure was conducted using the streptavidin‐peroxidase complex. The positive areas were outlined and then evaluated by a pathologist blinded to the experimental conditions as described previously.^33^

### 2.8 Electron microscopy

Transmission electron microscopy (TEM). Treated TECs were collected, centrifuged at 1000 *g* for 15 minutes and then fixed in 2.5% glutaraldehyde for 2 hours. The samples were then further fixed in 2% osmium tetroxide, dehydrated in an ascending alcohol series and embedded in Epon 812. Subsequently, ultrathin sections were cut and stained with uranyl acetate and lead citrate. The samples were observed under a transmission electron microscope (JEM 1210; JEOL, Ltd.).

Scanning electron microscope (SEM). TECs fixed with 2% osmium tetroxide were resuspended, concentrated, dropped on coverslips and allowed to adhere. The coverslips were then fixed and cut into correctly sized pieces. Images were viewed with a SEM (Hitachi S‐3400N).

### 2.9 Flow cytometric analysis

Flow cytometry was detected using a FACSCalibur flow cytometer (BD Biosciences). FITC‐conjugated Annexin V and propidium iodide (PI) staining assay kits and ROS assay kits were purchased from Beyotime Biotechnology. For cell death detection, TECs were harvested and then stained in the dark with Annexin V‐FITC and PI for 15 minutes prior to analysis. For the ROS analysis, the collected cells were cultured with the dichlorofluorescein diacetate (DCFH‐DA) cell‐permeant probe in the dark for 20 minutes and then transferred to the machine.

### 2.10 CaOx crystal‐cell adhesion assay

UV light‐illuminated CaOx crystals (100 μg/mL) were added to the cell culture dishes and then incubated at 37°C for 1 hours. Subsequently, the cells were vigorously washed with phosphate buffer saline (PBS) to eliminate unbound CaOx crystals. Finally, the adherent crystals on the cell surface were counted in 15 random high‐power fields under a phase‐contrast microscope (Eclipse Ti‐S; Nikon) as described previously.^34^

### 2.11 CaOx crystal internalization assay

For crystal internalization, high concentrations of FITC‐labelled (Thermo Scientific) CaOx crystals (500 μg/mL) were added at 37°C for 1 hour as mentioned above. The unbound crystals were washed out with PBS, and the cells were incubated with 5 mmol/L ethylene diamine tetraacetic acid (EDTA) in PBS to eliminate the adhered but uninternalized crystals prior to collection. The cells with internalized FITC‐labelled crystals were then quantified by flow cytometry as described previously.^30^

### 2.12 Ultrasound

An in vivo evaluation of renal calculus formation, cortical thickness and renal perfusion was conducted using an ultrasound system (iU Elite; Philips Healthcare) with B‐mode ultrasonography, USE and CDFI as the specific imaging modalities, respectively.

### 2.13 Clinical studies

Image‐diagnosed unilateral or bilateral renal calculus patients (n = 45) were studied. The inclusion criteria were as follows: (a) no anti‐inflammatory treatment before admission; and (b) no calculus in other regions. Pregnant women and patients with recent infections, autoimmune or metabolic disorders, malignant tumours, or severe organ or cardiovascular diseases were excluded. Healthy subjects who received checkups (n = 45) at the hospital during the same period were used as controls. Blood samples were subjected to anticoagulant and anti‐enzymatic treatment for the extraction of plasma as described previously and were then assayed using a relaxin‐3 ELISA Kit (KA5101; Abnova).^27^ Related serological indicators were assessed through routine biochemical analysis. The subjects provided informed consent. Prior to study initiation, the study complied with the Declaration of Helsinki and was approved by the Ethics Committee of the First Affiliated Hospital of Harbin Medical University.

### 2.14 RXFP‐1 knockdown

RXFP‐1 silencing was achieved through the transfection of specific siRNAs (sc‐40177; Santa Cruz Biotech) with Lipofectamine 2000 reagent (Invitrogen). The transfected TECs were recovered in complete DMEM for 4 hours and then cultured for 48 h prior to the subsequent examination or treatment. After Western blot verification, we selected the best siRNA for the subsequent experiments (Figure S6; Table S1).

### 2.15 Tandem Mass Tag™ labelling quantitative proteomics

Tandem Mass Tag^TM^ (TMT)‐related reagents and kits for isobarically label proteins of the TECs were selected to decrease the missing quantitative values (Thermo Scientific). Protein extraction, digestion and peptide labelling were sequentially performed (Figures S1 and S2A). Briefly, 100 μg of lytic protein per sample was reduced, alkylated, precipitated, resuspended and digested with trypsin before labelling.^35^ Subsequently, 20 μL of TMT was added to each sample and mixed, and sodium deoxycholate clearing and peptide desalting were performed.^36^ The pooled TMT‐labelled peptides were then separated and analysed using a reversed‐phase high‐performance liquid chromatography and a nano‐UPLC (EASY‐nLC 1200) coupled to a Q‐Exactive mass spectrometer (Thermo Finnigan) as previously described.^37^ The files were processed and quantitatively analysed using MaxQuant software (version 1.5.6.0), and the protein sequence database (UniProt, human, 2018, 10) was downloaded from UniProt.^36^, ^38^ Proteins with multiple relationships (ratio A/B > 1.25) and an adjusted *P* < .05 were defined as those with significant differences.

### 2.16 Bioinformatic analysis

After the differentially expressed proteins (DEPs) were screened, a hierarchical clustering analysis was performed using Cluster 3.0 and Java TreeView software. The DEPs were then annotated using the Database for Annotation, Visualization and Integrated Discovery (DAVID; https://david.ncifcrf.gov). Gene Ontology (GO) enrichment and Kyoto Encyclopedia of Gene and Genome (KEGG) pathway enrichment analyses were further performed based on Fisher's exact test to map the DEPs.^38^, ^39^ The protein‐protein interaction (PPI) networks were predicted using STRING (https://string-db.org) and Cytoscape software.^40^

### 2.17 Statistical analysis

The data are presented as the means ± SEMs derived from three repeated and independent experiments. One‐way ANOVA was used to analyse the differences among multiple groups, and a two‐tailed t test was used for comparisons between two groups. The associations between serum relaxin‐3 and other variables were evaluated using the Spearman correlation test. A *P*‐value less than .05 was considered statistically significant.

## 3 RESULTS

### 3.1 Proteomic analysis indicates that CaOx crystal‐induced TEC damage is closely related to the inflammation‐induced pyroptosis pathway

**FIGURE 1:**
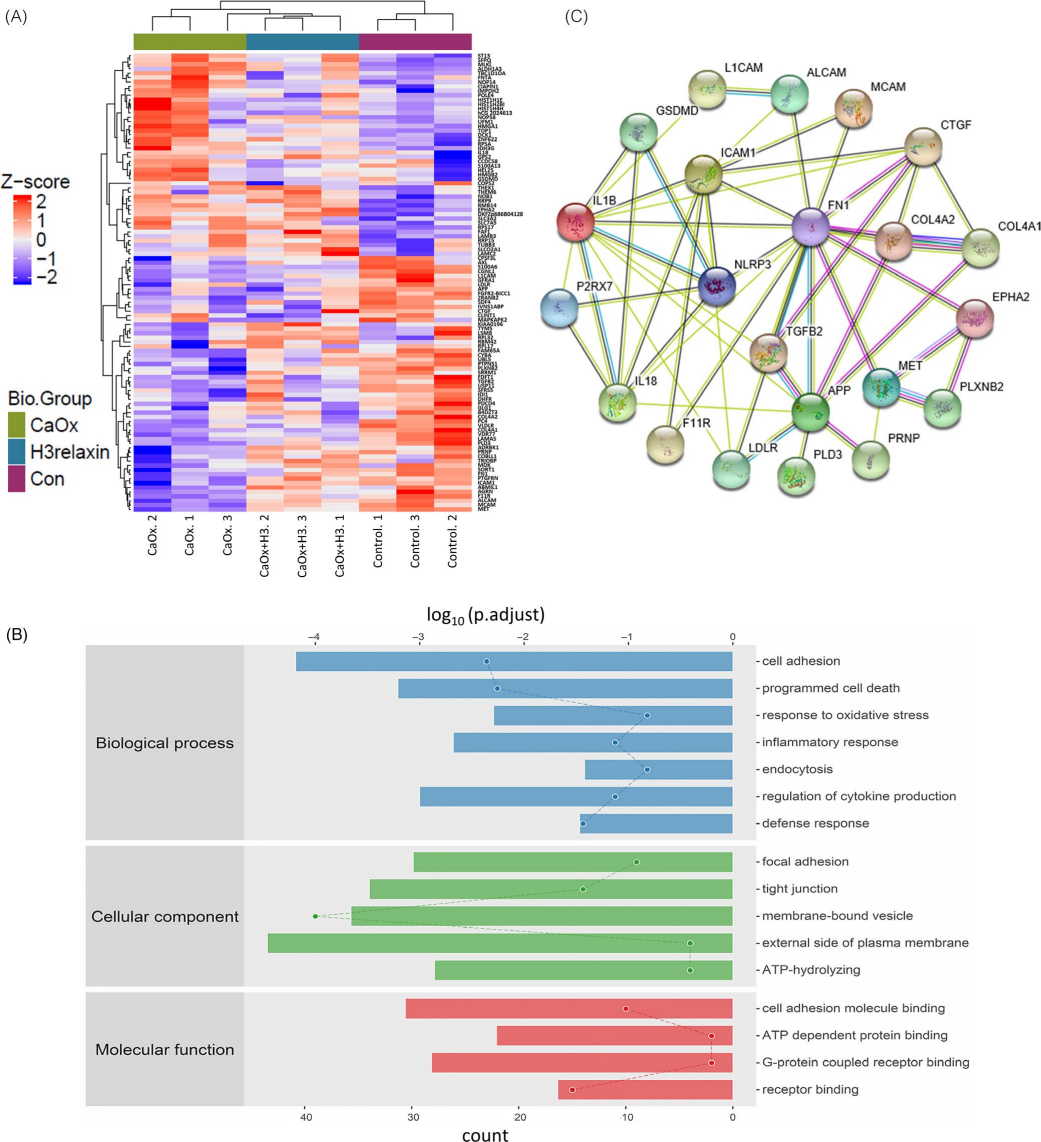
Proteomic analysis indicates that the CaOx crystal‐induced tubular epithelial cell (TEC) damage is closely related to the inflammation‐induced pyroptosis pathway. A, Hierarchical clustering of 104 differentially expressed proteins (DEPs) from the control and the CaOx crystal‐treated TEC samples in the absence or presence of H3 relaxin. Red represents increased expression, blue represents decreased expression, and white depicts no change in expression. n = 3, *P* < .05. B, The 104 DEPs were translated into significant biological properties using Gene Ontology enrichment analysis. The enriched biological behaviours were listed based on biological process, cellular component and molecular function. *P* < .05. C, A protein node complex composed of 22 significantly detected or inflammation‐related proteins, as a protein‐protein interaction network, was constructed by STRING online database. Only significant correlations are shown (*P* < .05)

To fully elucidate the effects of CaOx crystals on TECs and the effect of H3 relaxin, we performed liquid chromatography mass spectrometry‐based TMT labelling quantitative proteomic analyses. A total of 104 significant proteins were selected after detection and subjected to hierarchical cluster analysis, and many inflammatory factors, including GSDMD and IL‐18, were identified (Figure 1A). Subsequently, we performed GO and KEGG enrichment analyses to translate the high‐throughput data into biological properties. A series of inflammatory pyroptosis‐related biological behaviours were enriched, and these included the inflammatory response, programmed cell death, response to oxidative stress and membrane‐bound vesicle (Figure 1B; Figure S2B). And these biological processes are closely related to molecules such as NLRP3 inflammasome, caspase‐1, IL‐18, IL‐1β and GSDMD, which are the core of the inflammatory response pathway.^15^, ^18^ Additionally, other categories, such as cell adhesion, endocytosis and ATP hydrolysis, were also identified. Among the identified processes, the NLRP3 inflammasome‐centred inflammatory response pathway was selected because it is closely related to other detected pathways and closely linked to organ injury with clinical manifestations. Based on the selected pathway and the detected proteins, to better understand the regulatory mechanism of inflammatory pathway, a PPI network was further established using the STRING online database, and we then discovered a protein node complex centred on inflammatory pyroptotic components, as shown in Figure 1C. However, although damage to inflammatory pyroptosis has been validated by proteomics, some inflammatory proteins failed to be detected due to the sensitivity limitations of high‐throughput methods, and low‐throughput methods are thus needed to fully elucidate the complete process.

### 3.2 CaOx crystals induce renal pyroptosis in vivo/in vitro

**FIGURE 2:**
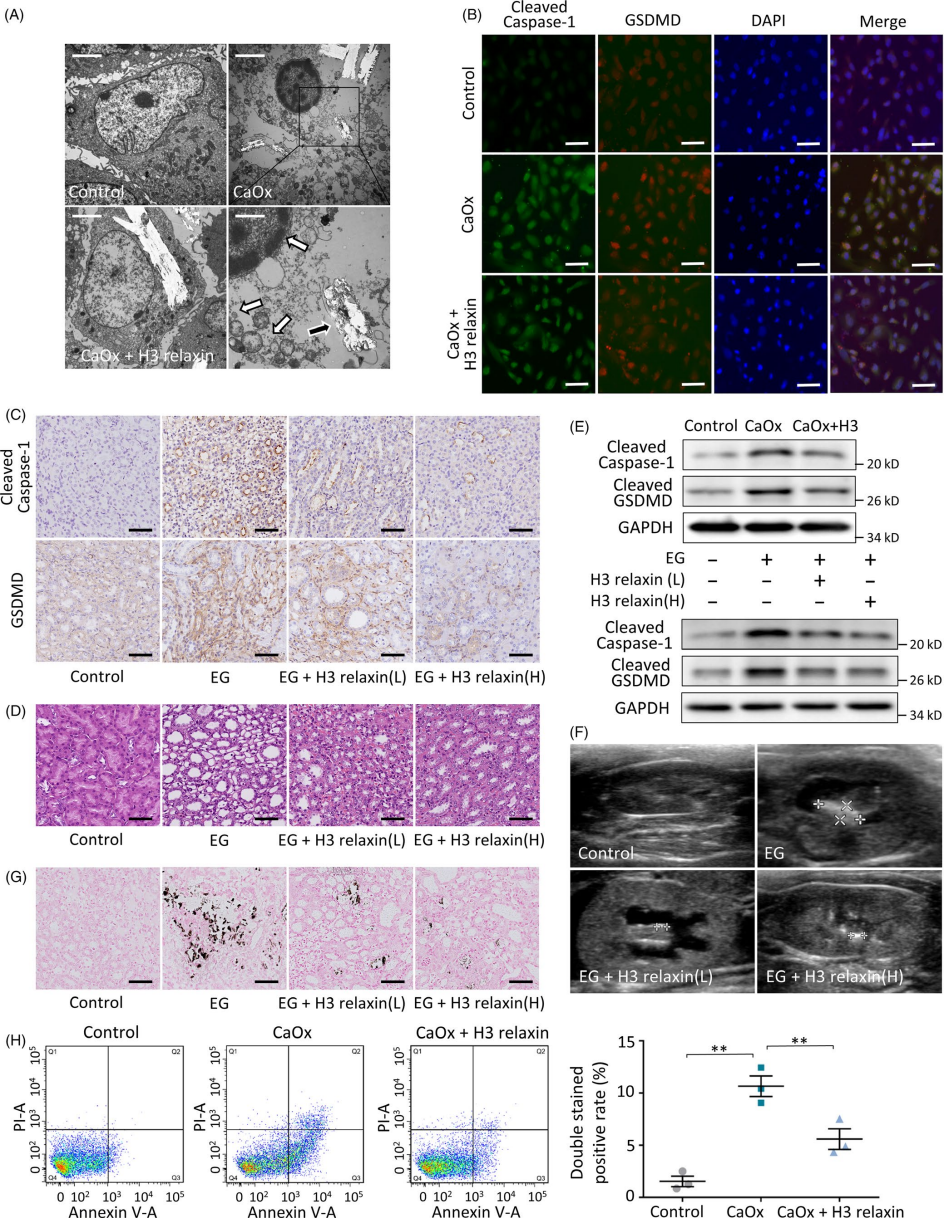
H3 relaxin improves the CaOx crystal‐related inflammatory pyroptosis in vivo/in vitro. A, tubular epithelial cells (TECs) were stimulated with CaOx crystals (0.5 mmol/L, 24 h) with or without H3 relaxin pretreated (20 ng/mL). Transmission electron microscopy (TEM) showed that swelling cell took up crystals (black arrow), pyknotic nuclei with chromatin margination (white arrow), vacuoles formed then spilled out (white arrows), whereas H3 relaxin treatment inhibited this performance. Scale bars: 10 μm (left, upper right); 3 μm (bottom right). B, The protein expression of cleaved caspase‐1 and cleaved gasdermin D was detected using immunofluorescence assays in the treated TECs in groups. Scale bars: 100 μm. C, The expression of same proteins was detected using immunohistochemical assays in kidneys of rats fed normal or ethylene glycol and NH_4_Cl added drink for 14 d, with or without H3 relaxin treated. Scale bars: 50 μm. D, Haematoxylin and eosin staining showed tubular dilatation; neutrophil infiltration; and epithelial cell deformation and exfoliation after 14 d of treatments. H3 relaxin improved these in a dose‐dependent manner. Scale bars: 50 μm. E, Western blot analysis of indicated proteins in the treated TECs or rats kidney samples (14 d), in the absence or presence of H3 relaxin. n = 3 per group, representative images are shown. F, Ultrasonographic examination showed CaOx crystal formation, hydronephrosis and renal cortex thinning in rats after 14 days replaced drinking, whereas H3 relaxin treatment inhibited this performance. G, Von Kossa staining also demonstrated obviously crystal deposition after 14 d of treatments, which were improved by H3 relaxin injection. n ≥ 3 per group, representative images are shown. H, Cell death detection of TECs after different treatments. n = 3, Data expressed as means ± SEM. *P < .05, **P < .01

Based on the proteomic results, we further examined the associated histopathological changes. The TEM observations showed that after 48 hours of exposure to CaOx crystals (0.5 mmol/L), the cells occasionally engulfed and took up the crystals (Figure 2A). In addition, the images showed swelling cells with sparse cytoplasm, pyknotic nuclei with chromatin margination, opened pores in the cell membrane and vacuoles, which spilled out into the extracellular matrix once the cells ruptured. These ultrastructural changes are typical morphological features of pyroptosis.^15^ In contrast to apoptosis, the main trigger of inflammatory pyroptosis has been confirmed to be caspase‐1‐cleaved GSDMD.^14^ To confirm that the observed loss of TEC membrane integrity is a result of pyroptosis, we visualized the expression and localization of cleaved caspase‐1 and GSDMD in treated TECs, and an immunofluorescence analysis revealed the increased levels of these proteins (Figure 2B). To further confirm the existence of this lesion in vivo, we duplicated the crystal‐related pathological process in rats. Immunohistochemical staining of the harvested kidneys revealed that the expression of pyroptosis‐related proteins increased significantly (Figure 2C; Figure S3A), which confirmed our hypothesis. A morphological analysis based on HE staining revealed substantial damage mainly at the inner stripe of the outer medulla: tubular oedema; diffuse neutrophil infiltration; and epithelial cell swelling, deformation, necrosis and exfoliation (Figure 2D; Figure S3B). Together, these observations indicate that CaOx crystals are associated with TEC pyroptosis in vitro and renal pyroptotic injury in vivo.

### 3.3 H3 relaxin improves CaOx crystal‐related inflammatory pyroptosis

As a widely accepted and organism‐derived polypeptide hormone, synthetic H3 relaxin has promising anti‐inflammatory and antioxidative activities.^41^ We first hypothesized that this hormone exerts a renal protective effect in our crystalline model. To investigate the functional effects of H3 relaxin in vitro, we pretreated CaOx crystal‐induced TECs with H3 relaxin (20 ng/mL) for 2 hours. The H3 relaxin‐treated cells showed strong improvements with less swelling, fewer internalized crystals and fewer pyroptotic cells compared with the CaOx crystal group, as observed by TEM (Figure 2A). Immunofluorescence assays revealed that H3 relaxin treatment decreases the levels of cleaved caspase‐1 and GSDMD, particularly in the cytoplasm (Figure 2B), and a Western blot analysis showed the same patterns for these proteins (Figure 2E; Figure S7A). Additionally, Annexin V‐FITC/PI staining showed that the intervention of CaOx crystals significantly increased the proportion of double‐stained positive TECs (Figure 2H). This cell death characterized by cell membrane perforation is consistent with cell pyroptosis, and H3 relaxin still exerted a significant protective effect. For further in vivo verification, rats were subcutaneously administered H3 relaxin in different doses, and renal histological analyses yielded results that were consistent with the above‐described findings: HE staining showed low‐grade dilatation of the tubules with decreased levels of mononuclear cell infiltrates in the interstitium (Figure 2D), and immunohistochemical assays showed decreased amounts of cleaved caspase‐1 and GSDMD in the experimental group (Figure 2C). For the comprehensive evaluation of kidney activity in vivo, we performed ultrasonographic examination of the bilateral kidneys before sacrifice. Although the CaOx crystals did not significantly affect organ perfusion, the crystal deposits increased in a dose‐ and time‐dependent manner, and this deposition leads to hydronephrosis and thinning of the renal cortex after 2 weeks of treatment. H3 relaxin injection relieved these symptoms, as shown in Figure 2F and Figure S4B. Von Kossa staining of isolated kidney tissues further confirmed similar differences in crystal formation (Figure 2G; Figure S4A). These data suggest that CaOx crystal‐induced nephropathy is at least partially alleviated by H3 relaxin in vivo and in vitro.

### 3.4 H3 relaxin inhibits GSDMD synthesis and pyroptosis by regulating NLRP3 inflammasome activation

**FIGURE 3:**
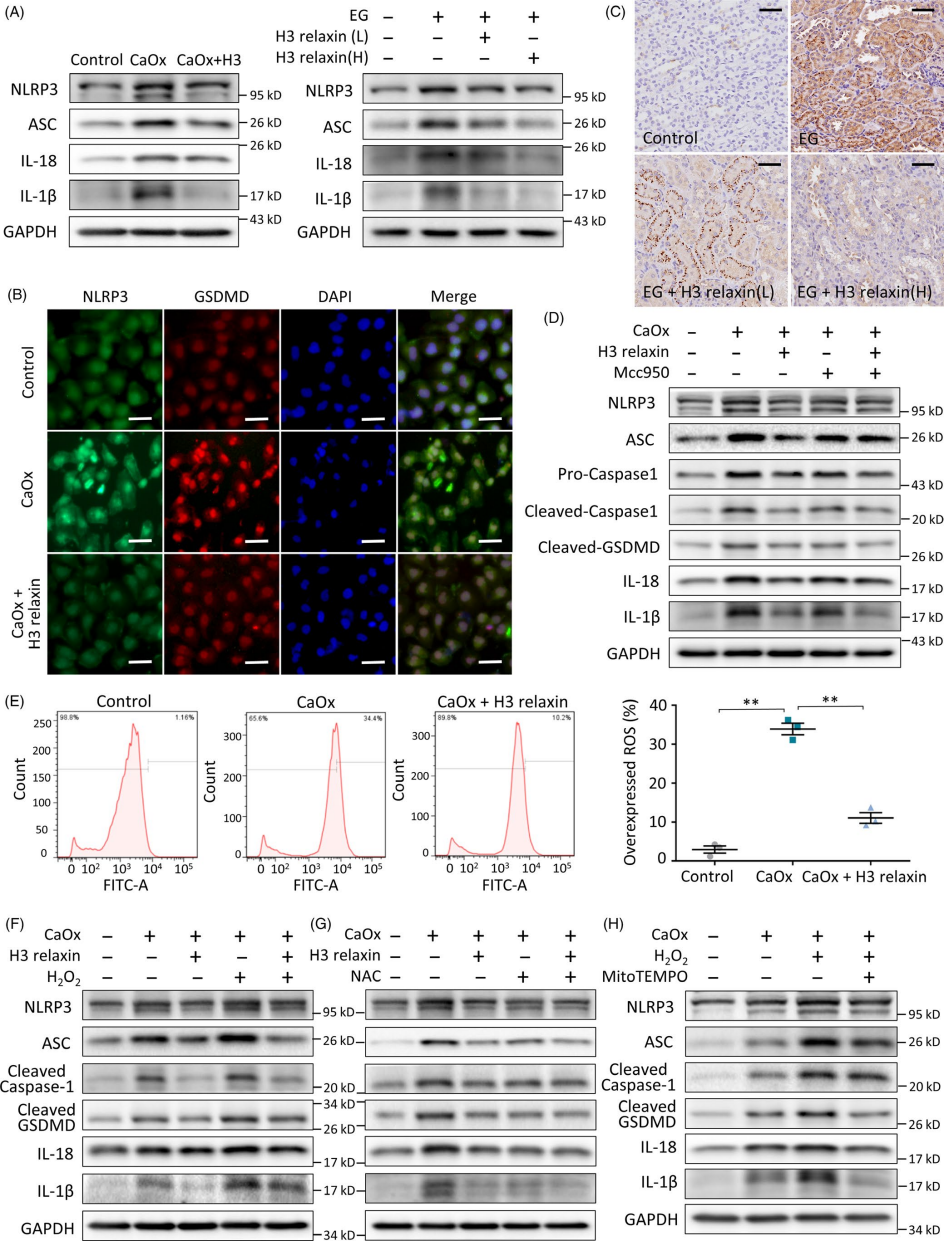
H3 relaxin inhibits CaOx crystal‐induced pyroptosis by preventing the reactive oxygen species (ROS)‐mediated NLRP3 inflammasome activation. A, Western blot analysis of related proteins: NLRP3, ASC, IL‐18 and IL‐1β in the treated tubular epithelial cells (TECs) or rat kidney samples (14 d), in the absence or presence of H3 relaxin. B, The protein expression of NLRP3 and cleaved GSDMD was detected using immunofluorescence assays in the treated TECs in groups. Scale bars: 100 μm. C, The protein expression of NLRP3 was detected using immunohistochemical assays in the treated rat kidneys (14 d), with or without H3 relaxin treated. Scale bars: 50 μm. D, Western blot analysis of inflammation‐related proteins: NLRP3, ASC, cleaved caspase‐1, cleaved GSDMD, IL‐18 and IL‐1β in the treated TECs, in the absence or presence of H3 relaxin and Mcc950. n = 3 per group, representative images are shown. E, ROS generation of the treated TECs was detected by DCFH‐DA probe using flow cytometric analysis. n = 3, data expressed as means ± SEM, **P < .01. F, Western blot analysis of inflammatory pyroptosis‐related proteins in the treated TECs, in the absence or presence of H3 relaxin and H_2_O_2_. G, Western blot analysis of inflammatory pyroptosis‐related proteins in the treated TECs, in the absence or presence of H3 relaxin and NAC. H, Western blot analysis of inflammatory pyroptosis‐related proteins in the treated TECs, in the absence or presence of H_2_O_2_ and MitoTEMPO. n = 3 per group, representative images are shown

The NLRP3 inflammasome was found to be a primary participant in the classical activation pathway of pyroptosis.^42^ We thus investigated its altered expression and relationship with caspase‐1 and GSDMD in CaOx crystal‐induced models. Our in vivo/in vitro examination of the constituent proteins of the NLRP3 inflammasome and the downstream proteins through Western blots revealed that H3 relaxin suppressed the CaOx crystal‐mediated increases in the NLRP3, ASC, IL‐18 and IL‐1β levels (Figure 3A; Figure S7B). This change in NLRP3 was further verified by immunofluorescence assays using TEC culture dishes and immunohistochemical assays using kidney sections (Figure 3B,C; Figure S3A). To further verify the regulatory roles of the NLRP3 inflammasome, we used M_CC_950, a specific inhibitor, which was added at a concentration of 1 μM. Western blot analysis showed a negative effect on the downstream proteins as predicted. However, in the M_CC_950 with H3 relaxin group, the effect of H3 relaxin was not obviously enhanced (Figure 3D; Figure S7C). This finding might be due to the specific inhibition of the NLRP3 inflammasome by M_CC_950, which results in inhibition of the inflammasome‐dependent anti‐pyroptosis effect of H3 relaxin. These data indicated that CaOx crystal‐induced GSDMD synthesis and pyroptosis are at least partially mediated by the NLRP3 inflammasome and might be inhibited by H3 relaxin. Notably, despite the reduced expression levels of cleaved caspase‐1, GSDMD, IL‐18 and IL‐1β observed in the M_CC_950 group, the NLRP3, ASC and pro‐caspase‐1 levels did not sharply decrease. Because the mechanism remains unclear, we hypothesized that M_CC_950 might suppress NLRP3 inflammasome activation instead of specific protein synthesis, and the definite target remains to be further explored.^43^

### 3.5 H3 relaxin inhibits CaOx crystal‐induced pyroptosis by preventing the ROS‐mediated NLRP3 inflammasome activation

Although ROS are considered a key factor that links cellular oxidative stress to NLRP3 inflammasome activation, the exact role of the redox state in these processes remains unclear.^42^, ^44^ Here, we attempted to elucidate the effect of ROS from different sources on inflammasome activation using our model. CaOx stimulated ROS generation, and the ROS‐mediated regulation of H3 relaxin was first proven by flow cytometry (Figure 3E). To determine the direct interaction between ROS and the NLRP3 inflammasome, we further treated the cells with an ROS agonist (H_2_O_2_, 1 mmol/L) or inhibitor (NAC, 10 mmol/L). H_2_O_2_ stimulation amplified the expression of the intracellular CaOx crystal‐related NLRP3 inflammasome (NLRP3, caspase‐1 and ASC) and downstream factors (GSDMD, IL‐18 and IL‐1β), and these effects were relieved by H3 relaxin (Figure 3F; Figure S8A). Furthermore, pretreatment with NAC substantially attenuated CaOx crystal‐induced protein upregulation (Figure 3G; Figure S8B). These combined effects indicate that GSDMD synthesis and pyroptosis are mediated by ROS‐induced NLRP3 inflammasome activation in CaOx crystal‐related injury and that these effects can be inhibited by H3 relaxin.

To further explore the mechanism underlying the ROS‐inflammasome interaction, MitoTEMPO, a specific mitochondrial ROS (mtROS) scavenger, was cocultured with CaOx crystal‐treated cells at a concentration of 20 μmol/L as described previously.^45^ Proteins downstream of the inflammasome were clearly negatively regulated, but the NLRP3 constituents showed only slight decreases, as indicated in Figure 3H and Figure S8C. Combined with relevant research, these findings indicate that the priming effect of ROS on NLRP3 leads to inflammasome formation and that mtROS is specifically and essentially needed for inflammasome activation.^46^

### 3.6 CaOx crystals trigger inflammatory pyroptosis via P2X_7_R‐mediated ROS activation

**FIGURE 4:**
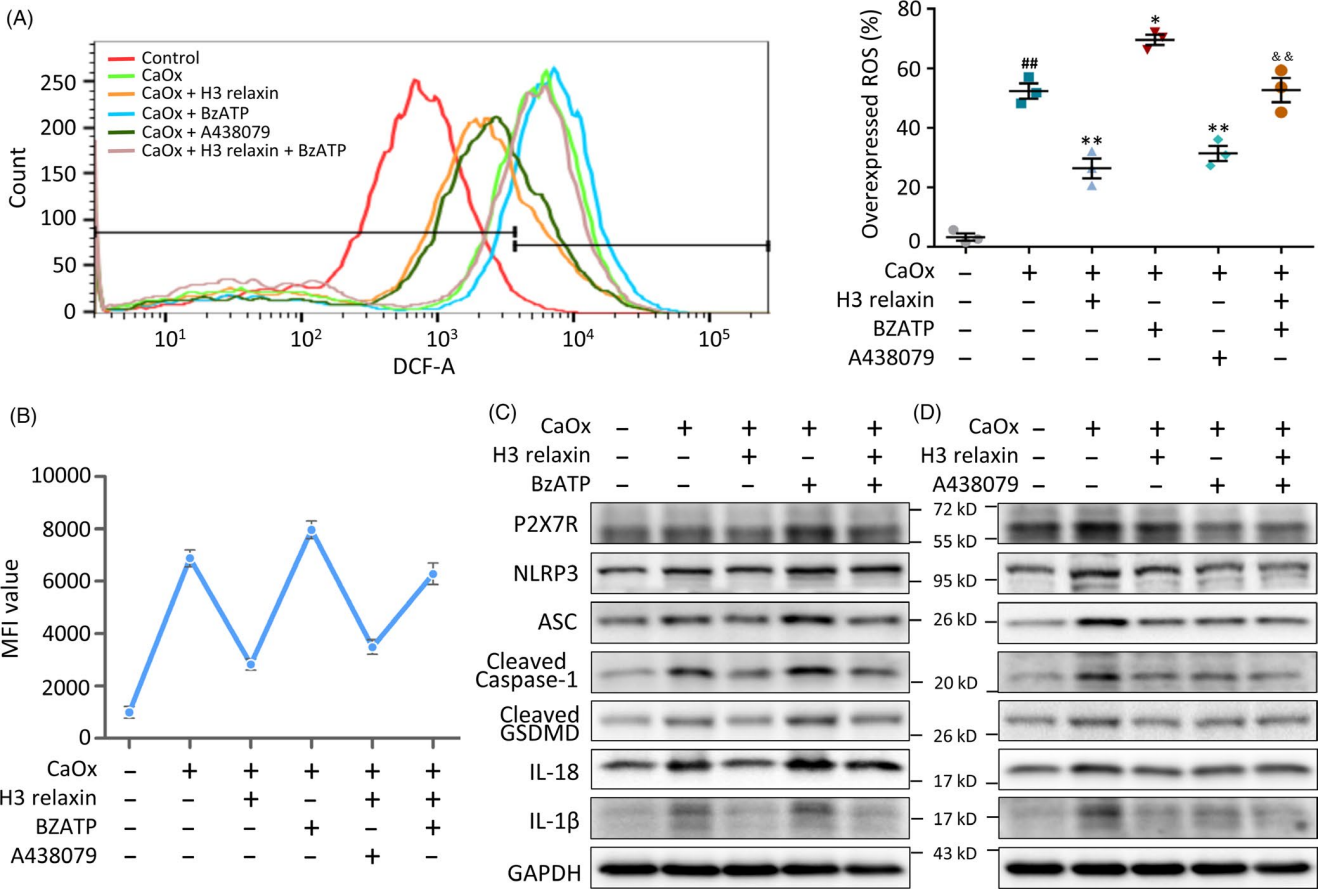
CaOx crystals trigger inflammatory pyroptosis via P2X_7_R‐mediated reactive oxygen species (ROS) activation. A, ROS generation of the treated tubular epithelial cells (TECs) was detected by DCFH‐DA probe using flow cytometric analysis, with or without the pretreatment of H3 relaxin, BzATP and A438079. n = 3, data expressed as means ± SEM, ^##^ *P* < .01 vs control group, *P < .05 vs CaOx group, **P < .01 vs CaOx group, ^&&^ *P* < .01 vs CaOx + BzATP group. B, Mean fluorescence intensity of the ROS detected TECs, as a supplement to the overexpressed ROS percentage in different groups. n = 3, data expressed as means ± SEM, *P* < .05, comparison principles unchanged. C, Western blot analysis of P2X_7_R and inflammatory pyroptosis‐related proteins in the treated TECs, in the absence or presence of H3 relaxin and BzATP. D, Western blot analysis of P2X_7_R and inflammatory pyroptosis‐related proteins in the treated TECs, in the absence or presence of H3 relaxin and A438079

Potassium (K^+^) efflux has been proven to be one of the necessary early events in ROS activation.^47^ We hypothesized that K^+^ channel‐coupled P2X_7_R might be related to ROS activation and further evaluated the role of this upstream signal through stimulation with CaOx crystals to activate pyroptosis. To specifically analyse the effects of P2X_7_R, we pretreated CaOx crystal‐treated TECs with/without a P2X_7_R agonist (BzATP, 10 mmol/L) or inhibitor (A438079, 10 mmol/L). A flow cytometry analysis indicated that P2X_7_R activity was positively correlated with ROS expression in the treated cells (Figure 4A,B). We further found that BzATP aggravated the crystal‐induced increases in the expression of NLRP3, ASC, cleaved caspase‐1 and the downstream factors GSDMD, IL‐18 and IL‐1β (Figure 4C; Figure S8D). In contrast, A438079 reversed these increases and might synergistically function with H3 relaxin, as shown in Figure 4D and Figure S9A. The above‐mentioned results suggest that CaOx crystal‐induced inflammatory pyroptosis is at least partially mediated by activation of the P2X_7_R‐ROS‐NLRP3 inflammasome pathway. Although P2X_7_R expression was altered by the addition of the agonist and inhibitor, its expression was not significantly changed after the addition of H3 relaxin (Figure 4C,D). This finding suggests that P2X_7_R expression is not altered by the antioxidative/anti‐inflammatory pyroptosis effects of H3 relaxin and that the latter might directly or indirectly act on ROS.

### 3.7 H3 relaxin relieves CaOx crystal‐induced cell damage by regulating eATP levels

**FIGURE 5:**
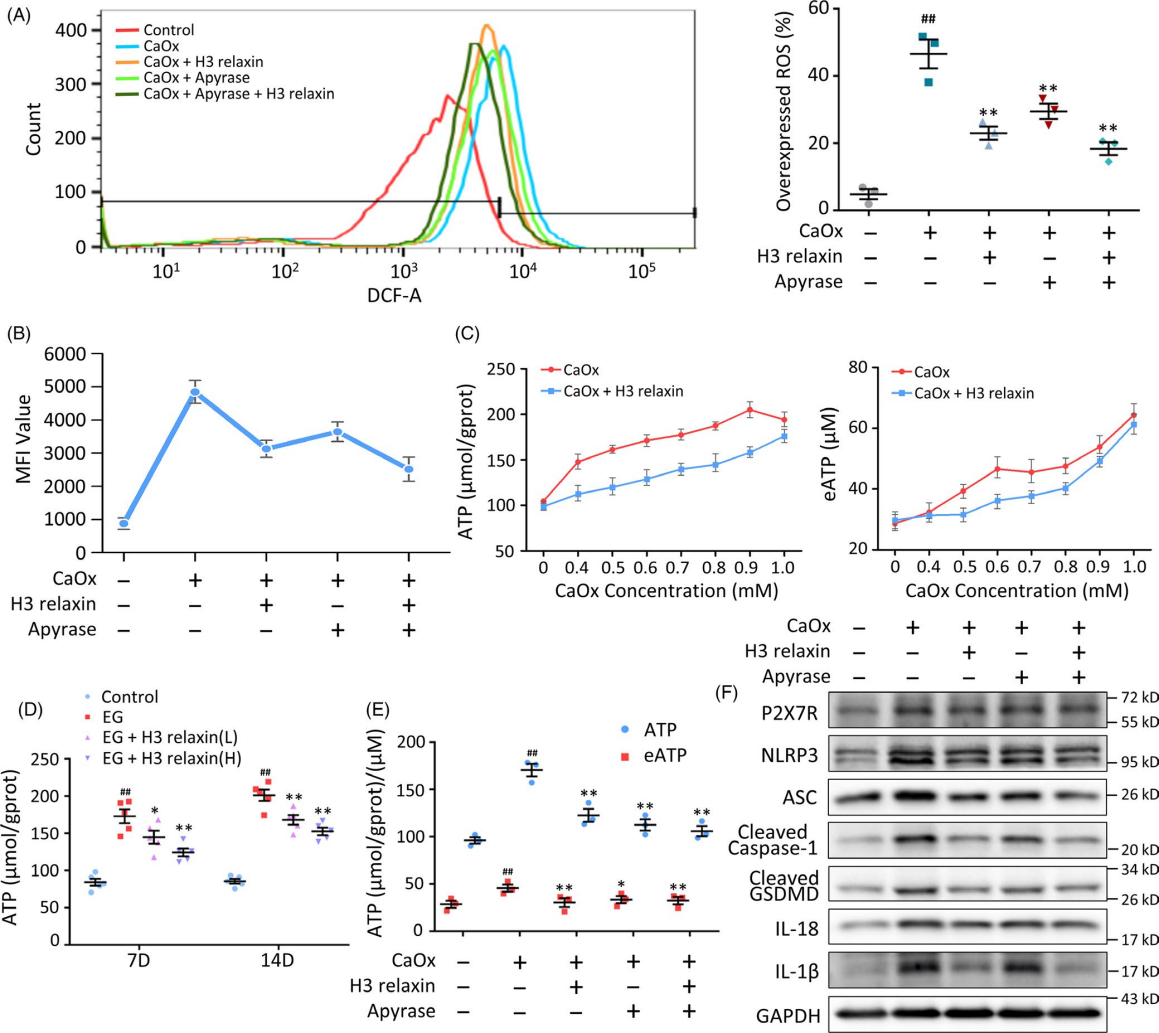
H3 relaxin relieves the CaOx crystal‐induced cell damage by regulating the extracellular ATP levels. A, Reactive oxygen species (ROS) analysis of the treated tubular epithelial cells (TECs) with or without the pretreatment of H3 relaxin and apyrase. n = 3, data expressed as means ± SEM, ^##^ *P* < .01 vs control group, *P < .05 vs CaOx group, **P < .01 vs CaOx group. B, Mean fluorescence intensity of the ROS detected TECs, as a supplement to the overexpressed ROS percentage. n = 3, data expressed as means ± SEM, *P* < .05, comparison principles unchanged. C, TECs were stimulated with increasing dose of CaOx crystals with or without H3 relaxin pretreatment. Intracellular ATP and eATP levels were measured using colorimetric ATP assay. n = 3, data expressed as means ± SEM, *P* < .05 control vs each point in CaOx group, *P* < .05 CaOx + H3 relaxin group vs CaOx group. D, ATP levels in renal tissue extracts of the treated rats (7, 14 d) were detected using colorimetric ATP assay. n = 5, data expressed as means ± SEM, ^##^ *P* < .01 vs control group, *P < .05 vs ethylene glycol (EG) group, **P < .01 vs EG group. E, Intracellular ATP and eATP levels in the treated TECs in the absence or presence of H3 relaxin and apyrase. n = 3, data expressed as means ± SEM, ^##^ *P* < .01 vs control group, *P < .05 vs CaOx group, **P < .01 vs CaOx group. F, Western blot analysis of P2X_7_R and inflammatory pyroptosis‐related proteins in the treated TECs, in the absence or presence of H3 relaxin and apyrase

Extracellular ATP activates P2X_7_R and triggers inflammasomes.^47^, ^48^ To verify the involvement of ATP in the induction of pyroptotic injury by CaOx crystals and its amelioration by H3 relaxin, we tested its levels in vivo and in vitro. The in vitro experiments showed that although the eATP content was markedly lower than the intracellular ATP content, both factors were significantly increased by CaOx crystals in a dose‐dependent manner and reduced by H3 relaxin (Figure 5C). This trend was confirmed in kidney tissue extracts from SD rats (Figure 5D). To further evaluate the regulatory role of ATP, apyrase (1 U/mL), a specific ATP inhibitor, was added before the cells were measured, and the results showed that the ATP (eATP) levels were clearly degraded (Figure 5E). Apyrase also exerted a significant negative effect on ROS and the proteins downstream of the NLRP3 inflammasome, as indicated in Figure 5A,B,F and Figure S9B. Consistent with our previous conclusions, P2X_7_R expression was not substantially changed by treatment with apyrase or H3 relaxin. We consequently hypothesized that the regulation of P2X_7_R functional activity is mediated through control of the coupled K^+^ channels but not the number of receptors. Thus, CaOx crystals might activate ATP, promote the release of eATP, decrease the intracellular K^+^ concentration and then activate ROS, leading to activation of the inflammatory pyroptosis pathway, and these effects might be inhibited by H3 relaxin.

### 3.8 H3 relaxin reduces ATP and ROS synthesis through the RXFP1‐cAMP pathway

**FIGURE 6:**
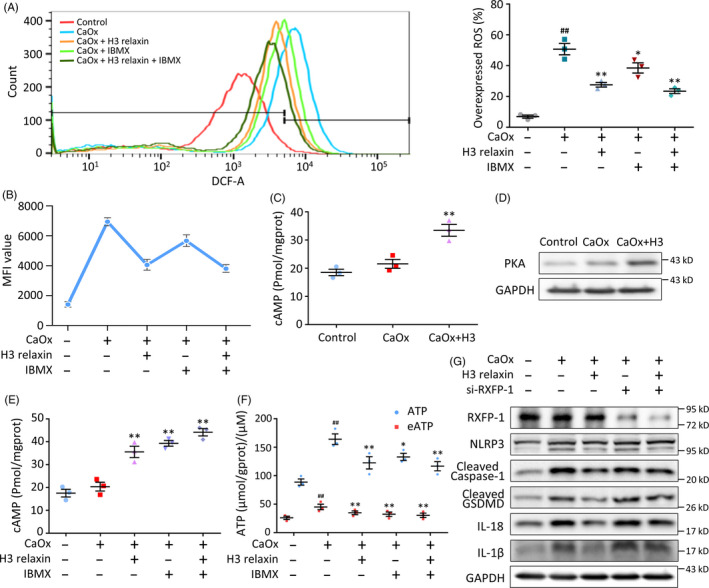
H3 relaxin reduces ATP and reactive oxygen species (ROS) synthesis through the RXFP1‐cAMP pathway. A, ROS analysis of the treated tubular epithelial cells (TECs) with or without the pretreatment of H3 relaxin and 3‐isobutyl‐1‐methylxanthine (IBMX). n = 3, data expressed as means ± SEM, ^##^ *P* < .01 vs control group, *P < .05 vs CaOx group, **P < .01 vs CaOx group. B, Mean fluorescence intensity of the ROS detected TECs, as a supplement to the overexpressed ROS percentage. n = 3, data expressed as means ± SEM, *P* < .05, comparison principles unchanged. C, cAMP levels of treated TECs were measured using enzyme‐linked immunosorbent assay. n = 3, data expressed as means ± SEM, **P < .01 vs control group. D, Western blot analysis of PKA in the treated TECs, in the absence or presence of H3 relaxin. n = 3, representative image is shown. E, cAMP levels in the treated TECs in the absence or presence of H3 relaxin and IBMX. n = 3, data expressed as means ± SEM, **P < .01 vs control group. F, Intracellular ATP and eATP levels in the treated TECs in the absence or presence of H3 relaxin and IBMX. n = 3, data expressed as means ± SEM, ^##^ *P* < .01 vs control group, *P < .05 vs CaOx group, **P < .01 vs CaOx group. G, Western blot analysis of RXFP‐1 and inflammatory pyroptosis‐related proteins in the treated TECs, in the absence or presence of H3 relaxin and si‐RXFP‐1 pretreatment. n = 3 per group, representative images are shown

RXFP1‐cAMP is an important factor in native and isolated cell lines that responds to relaxin.^49^ H3 relaxin binds to and then activates RXFP1 in addition to its unique receptor RXFP3.^50^ Based on antioxidant research of the cAMP pathway, we hypothesized that this molecule can regulate the balance of ROS in CaOx crystal‐induced cell damage. As detected by ELISA Kits and Western blotting, the cAMP and PKA levels were upregulated by H3 relaxin (Figure 6C,D). We used IBMX, a phosphodiesterase inhibitor that functions as an agonist of cAMP, to further verify the direct effect of cAMP on ROS. We first confirmed that IBMX upregulated cAMP, which was similar to the effect of H3 relaxin (Figure 6E). The intracellular ATP and eATP levels and the cellular ROS levels were decreased after IBMX or H3 relaxin treatment, and these two treatments exerted a relatively synergistic effect (Figure 6A,B,F). Moreover, the H3 relaxin‐mediated protection was blocked by the knockout of the RXFP‐1 receptor, which further confirmed the effect and target of this treatment (Figure 6G; Figure S9C). These combined data suggest that H3 relaxin eliminates excess ATP and ROS at least partially through the RXFP1‐cAMP pathway. Therefore, we proved that H3 relaxin might ameliorate CaOx crystal‐induced inflammatory pyroptosis via the RXFP1‐cAMP‐regulated ROS‐NLRP3 inflammasome‐GSDMD axis.

### 3.9 The anti‐inflammatory and antioxidative effects of H3 relaxin might alleviate crystal‐cell adhesion and crystal internalization

**FIGURE 7:**
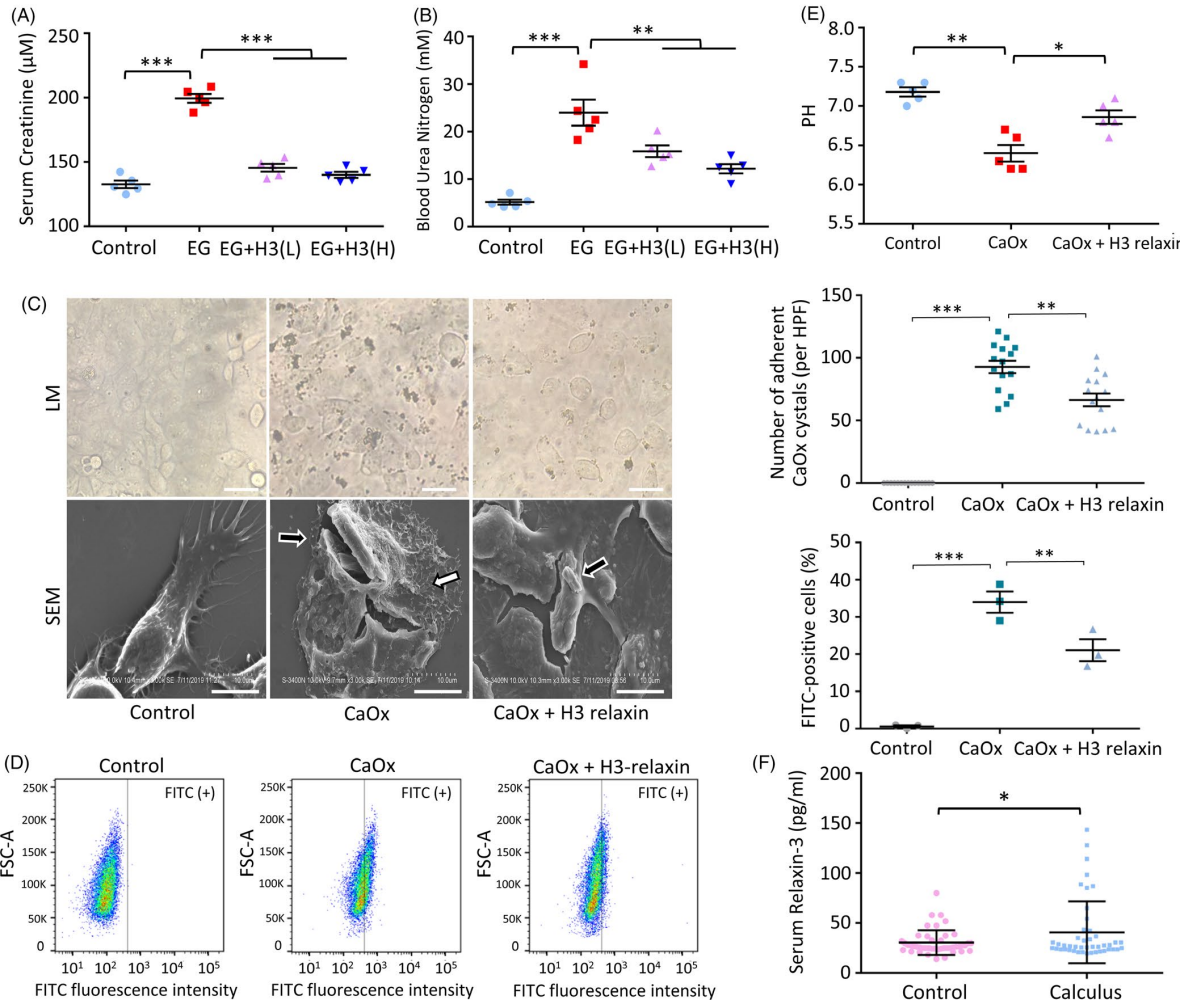
The anti‐inflammatory and antioxidative effects of H3 relaxin may relieve the crystal‐cell adhesion and the crystal internalization in vitro, and the crystal‐related renal function damage in vivo. A and B, Renal function of treated rats (14 d) was evaluated by creatinine and urea assays. n = 5 per group, data expressed as means ± SEM, **P < .01, ***P < .001. C, CaOx crystal‐cell adhesion assay was detected by light microscopy (LM) and scanning electron microscopy (SEM). Images under LM showed less adhered CaOx crystals per fields after H3 relaxin pretreated. n = 15, data expressed as means ± SEM, **P < .01, ***P < .001. Scale bars: 100 μm. Images under SEM showed obvious crystal adhesion (black arrow) and cell rupture (white arrow) in CaOx group, which will be inhibited by H3 relaxin. Scale bars: 10 μm. D, CaOx crystal internalization assay was detected by FITC probe using flow cytometric analysis. n = 3, data expressed as means ± SEM, **P < .01, ***P < .001. E, The cell medium pH level analysis of TECs. n = 3, data expressed as means ± SEM, *P < .05, **P < .01. F, Serum relaxin‐3 levels of healthy subjects and calculus patients. n = 45 per group, data expressed as means ± SEM, *P < .05

The physical damage caused by CaOx crystals might lead to TEC inflammatory pyroptosis. Through cell experiments, we also found that the treatment of CaOx crystal‐injured cells with H3 relaxin decreased the crystallization density. To further verify whether damaged cells are more likely to aggregate and take up CaOx crystals, which could result in a vicious cycle, we performed CaOx crystal‐cell adhesion and internalization assays. The data showed that H3 relaxin clearly decreased the adhesion between CaOx crystals and TECs (Figure 7C). The coculture of FITC‐labelled CaOx crystals with H3 relaxin‐pretreated cells resulted in a decreased fluorescence intensity, as demonstrated by flow cytometry (Figure 7D). In addition to the injury‐related unveiling of membrane lipids, the pH level of the cell medium influences the cell adhesion and endocytosis of crystals.^34^ We further found that H3 relaxin could neutralize the acidification of the culture media caused by the inflammatory damage induced by CaOx crystals (Figure 7E). We therefore concluded that the anti‐inflammatory and antioxidative effects of H3 relaxin might alleviate crystal‐cell adhesion and internalization by reducing cellular metabolic damage and regulating the environmental pH, and the in‐depth mechanism needs further study.

### 3.10 The anti‐inflammatory and antioxidative effects of H3 relaxin might relieve CaOx crystal‐related renal function damage in vivo

Relaxin allows the recovery of renal function through multiple mechanisms.^51^, ^52^ To further verify whether relaxin‐3 exerts this effect, we assessed serological markers representing kidney function using sacrificed animal serum. EG increased the Scr and BUN levels significantly, and both of these biomarkers were stably decreased after the injection of H3 relaxin (Figure 7A,B; Figure S5A, B). These changes demonstrate that H3 relaxin protects against the renal damage caused by CaOx crystals. Because CaOx crystal‐induced renal failure has been related to inflammatory injury as well as intratubular obstruction, as previously indicated, H3 relaxin may inhibit the effect of crystal deposition through its anti‐inflammatory action, as mentioned previously.^53^

### 3.11 A clinical study: Patients with renal calculus show increased relaxin‐3 plasma levels

Combined with previous studies, our results further elucidate the regulatory role of exogenous relaxin in inflammation.^21^ The self‐regulating effects of secreted endogenous relaxin should also be investigated. To determine the level of endogenous relaxin‐3 in patients with CaOx crystal‐related nephropathy and analyse its physiological effects, we studied various serological indicators as well as serum relaxin‐3 in patients and healthy subjects. Relevant serum indicators and demographic characteristics are shown in Table 1. The analysis showed that the relaxin‐3 levels were higher in the patients than in the controls (Figure 7F), which indicates that this molecule might be involved in a self‐protective reaction. Serum relaxin‐3 also showed significant associations with Scr, BUN and β2‐microglobulin, which are often regarded as biomarkers of renal function (Table 1), and this effect suggests the notable potential of serum relaxin‐3 as an early indicator of inflammatory injury.

**TABLE 1:** Clinical and biochemical characteristics of the subjects, and the correlation coefficients between serum relaxin‐3 levels with different parameters in the study groups

Variables	Controls (n = 45)	Renal calculus (n = 45)	Relaxin‐3 Correlation coefficient (*r*)
Age (y)	57 (25‐79)	55 (25‐76)	.000
Gender (male/female, n)	22/23	22/23	.070
BMI (kg/m^2^)	23.81 ± 3.43	24.01 ± 3.97	.002
Relaxin‐3 (pg/mL)	30.53 ± 12.46	40.78 ± 30.95*	—
Scr (μmol/L)	63.58 ± 12.38	97.59 ± 69.92**	.293**
BUN (mmol/L)	5.77 ± 1.20	6.87 ± 3.32*	.278**
Cys‐c (mg/L)	0.87 ± 0.14	1.17 ± 0.39**	.253*
β2‐MG (mg/L)	1.39 ± 0.38	2.40 ± 1.59*	.338**
RBP (mg/L)	43.06 ± 13.56	48.70 ± 14.42**	.239*

Note

Data expressed as means ± SEM, *P < .05 vs controls, **P < .01 vs controls. *r* = correlation coefficient, *P < .05, **P < .01.

Abbreviations: β2‐MG, β2‐microglobulin; Cys‐c, cystatin c; BMI, body mass index; BUN, blood urea nitrogen; RBP, retinol‐binding protein; Scr, serum creatinine.

## 4 DISCUSSION

**FIGURE 8:**
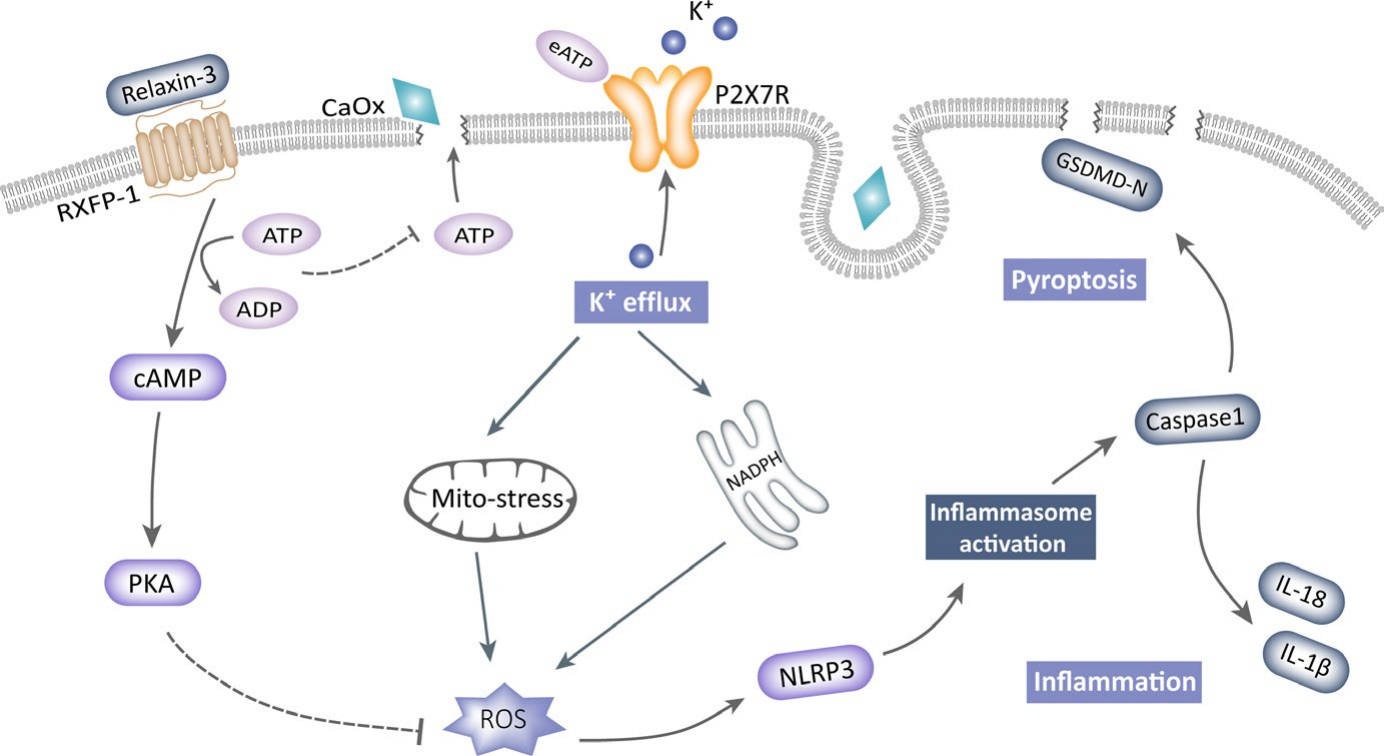
Model of H3 relaxin protects against calcium oxalate crystal‐induced renal inflammatory pyroptosis

Macroscopic intraluminal obstruction was considered a pathogenic factor and therapeutic focus for urolithiasis due to its visibility and specificity until the refractory nature of nephrocalcinosis and the recurrence of renal stone disease after surgery led to a reevaluation of the methods used to protect renal function.^54^, ^55^ The NLRP3 inflammasome axis is known to trigger cell damage and organ dysfunction in many fields, including crystalline nephropathy.^48^ Based on previous theories and our new morphologically supported findings, we elucidated an intact inflammatory pyroptotic pathway that links CaOx crystals to renal impairment. We further validated the significance of the endogenous and exogenous hormone relaxin‐3 in the prevention of CaOx crystal‐induced inflammation, which will aid the development of anti‐inflammatory strategies for clinical applications (Figure 8).

Previous explorations of inflammatory pyroptotic mechanisms have mostly examined immunocytes such as macrophages and dendritic cells due to their high abundance of inflammatory mediators with typical characteristics.^46^, ^48^ Due to the universality of cellular inflammatory reactions and regulations, the role of pyroptosis in nonimmune cells, including epithelial, endothelial and interstitial cells, has gradually been recognized, and this study provides the first elucidation of this CaOx crystal‐sensing mechanism in TECs.^19^, ^56^, ^57^ The results of this study regarding the altered phenotype and protein expression of TECs in vivo and in vitro clearly demonstrate that immunocytes are not the only participants in this inflammatory pyroptotic process. In fact, as the first sensor of crystals, TECs induce an active, not just auxiliary, inflammatory response. We first performed a high‐throughput proteomic analysis to verify the inflammatory damage induced by CaOx crystals on TECs, evaluate the counteracting effects of H3 relaxin and identify the key‐enriched pathways. Changes in multiple pathways, including the inflammatory response, intercellular adhesion and ATP hydrolysis, were found to affect the biological behaviour of TECs under intervention. We believe that inflammation‐related pathways have the strongest clinical relevance and research value for the extensive communication among inflammatory factors. Although important inflammatory or pyroptotic executors were found to be significant, several members of the target axis failed to be detected due to their low levels and the limited sensitivity of high‐throughput methods.^58^ We therefore speculated that in addition to proteomics, classical but highly sensitive low‐throughput protocols might be needed for the detection of TEC inflammation and should be performed.

Using M_CC_950, an NLRP3 inflammasome inhibitor, we proved that the inflammasome‐activated effector caspase‐1 promotes pro‐IL‐1β, pro‐IL‐18 and GSDMD maturation and that this effect is also observed in CaOx crystal‐treated TECs.^43^ We further confirmed that the amplification and activation of the NLRP3 inflammasome are positively regulated by ROS in our model using the ROS agonist H_2_O_2_ and inhibitor NAC, which is consistent with previous findings showing that ROS promote inflammatory mediators through the NF‐κB pathway.^59^ Notably, intracellular ROS have multiple sources, and priming and activation of the NLRP3 inflammasome are two consecutive but differently regulated processes.^10^, ^60^ In our study, we used MitoTEMPO to block mitochondrial‐derived mtROS, and the analysis showed that the factors downstream of the inflammasome were sharply decreased even though its components showed only a slight reduction. Based on these results together with the finding that mtROS‐modified ox‐mtDNA participates in the assembly of the NLRP3 inflammasome, we speculate that although ROS from multiple sources can lead to priming, mtROS play an essential role in activation and might be a potential target for limiting this particular damage without compromising host defence.^46^ The above‐mentioned components, including ROS, were decreased by H3 relaxin, which indicates that its target might be located upstream of the inflammasome.

How can CaOx crystals elevate ROS in TECs? Pathogen invasion, particle uptake, lysosomal rupture, K^+^ efflux and exogenous ROS entry all contribute to increased intracellular ROS levels through NADPH and the mitochondrial pathway, and K^+^ efflux is particularly effective due to its relatively low threshold and universality.^46^, ^59^, ^61^ P2X_7_R, a transmembrane ligand‐gated K^+^ channel that exhibits a rapid response to eATP, is expressed in TECs, and its activity is closely associated with activation of the NLRP3 inflammasome.^47^, ^62^ Using the P2X_7_R‐specific agonist BzATP and the inhibitor A438079, our study proved that the intracellular ROS levels were linearly regulated by P2X_7_R activity, and the same trend was found for the NLRP3 inflammasome and downstream cytokines. Based on previous studies and the characteristics of transmembrane proteins, we further proposed that P2X_7_R‐controlled K^+^ efflux might affect the NLRP3 inflammasome axis via ROS regulation. Notably, in addition to the P2X_7_R‐coupled pathway, pyroptosis‐induced membrane rupture is considered another channel of K^+^ outflow, and the inflammatory procedure might be further aggravated to form a vicious cycle.^12^, ^63^ Coincidentally, recent research has revealed that chronic eATP dosing will transform this triggered membrane channel into a large opened pore; thus, P2X_7_R can induce membrane damage through more than one mechanism.^64^ By detecting the ATP levels in extracellular fluids and verifying these findings with the ATP hydrolase apyrase, we also concluded that the adhesion of crystals activates P2X_7_R and subsequently downstream reactions in an ATP‐dependent manner in TECs. However, the ATP level did not affect the expression of P2X_7_R itself, which indicates that ATP stimulates its activation but not its abundance.

How can CaOx crystals induce increases in the eATP level in TECs? In general, eATP can be derived from intracellular and extracellular sources, mainly through its release from bacteria or surrounding ruptured cells.^47^ Because intracellular ATP is not free to cross the membrane, healthy interstitial eATP levels are fixed in the nanomolar range and are tightly regulated by ubiquitous extracellular nucleotidases in the case of cascade activation caused by excess levels.^64^ However, the eATP concentrations around inflammatory or damaged cells are increased by several‐fold, although the specific mechanisms are complicated.^65^ Moreover, studies have shown that in the inflammatory environment, CD44, which was also detected in TECs through our proteomic analyses, positively modulates P2X_7_R by facilitating eATP binding.^66^ Cell rupture has long been regarded as the main mechanism underlying the release of cellular ATP, and until now, it was believed that important nonlytic release frequently occurs via several mechanisms under certain conditions, including ATP‐binding cassette transporters, gap junctions, vesicular exocytosis and P2X_7_R.^62^, ^65^ These combined mechanisms along with direct physical damage caused by CaOx crystals might explain the elevated eATP levels observed in our models. Additionally, oxidative stress and inflammation induced by crystals will damage the mitochondrial respiratory chain and cause cellular ATP accumulation, providing potential energy for its release. We thus described the complete process of this injury.

How, then, can H3 relaxin relieve the above‐described damage? Although the anti‐inflammatory and antioxidative functions of H3 relaxin have been studied in various diseases, few studies have reported its specific targets. According to pharmacological views, the RXFP1‐cAMP axis is the main pathway mediating the biological effects of the relaxin family; relaxin increases the levels of cAMP and downstream PKA by acting on RXFP1, as was verified in our H3 relaxin‐treated TEC model.^22^, ^50^, ^67^ The synthesis of cAMP requires ATP, and our proteomic study showed ATP hydrolytic activity in the H3 relaxin‐protected group. We then hypothesized that activation of the RXFP1‐cAMP axis attenuates inflammatory responses by consuming ATP.^68^, ^69^ To test this hypothesis, we used the cAMP functional agonist IBXM to induce decreases in ROS and ATP and found that this agonist exerted a synergistic effect when combined with H3 relaxin, which indicates that this ATP‐to‐cAMP transformation process is at least one of the anti‐inflammatory targets of H3 relaxin. In addition, the relaxin family reduces ROS by decreasing NADPH activity and increasing endogenous superoxide dismutase, whereas the promoted cAMP pathway can also reduce ROS by upregulating PI3K/Akt signalling.^70^, ^71^, ^72^ However, although we have revealed the possible mechanisms of H3 relaxin‐mediated protection against this inflammatory pyroptotic injury, its multiple beneficial effects indicate that its mechanism might not simply be limited to this particular target or even the anti‐inflammatory pathway, and further exploration is needed.

What is the relevance of our results to clinical practice in CaOx crystalline nephropathy? First, the described mechanisms, from crystal adhesion to the expression of inflammatory pyroptotic factors, provide a range of potential targets for limiting this clinical renal damage. Second, based on the observed effects of H3 relaxin on the pH of the culture media, crystal adhesion to TECs and crystal internalization, we hypothesized that anti‐inflammatory agents with antioxidative effects might reduce crystal deposition and even stone formation by improving the microenvironment of epithelial cells, which provides a novel theoretical basis for urine alkalization in renal calculus treatment. Third, we performed a clinical serological test and found increased plasma levels of relaxin‐3 in patients with renal calculus, which is consistent with previous studies on endogenous relaxin in other diseases.^22^, ^49^ These differences not only support our results that this hormone participates in the regulation of inflammation in vivo but also reveal its potential as a systemic inflammatory biomarker. However, the sensitivity to inflammation in different organs requires further elucidation, and gender differences combined with pregnancy‐secreted characteristics might limit its clinical application.

In summary, we observed a new form of cellular damage in TECs and elucidated a complete inflammatory pyroptotic mechanism that promotes stone formation and decreased renal function. This CaOx crystal‐triggered increases in the P2X_7_R‐ROS‐NLRP3 inflammasome axis are required for the activation of TEC pyroptosis, which suggests several therapeutic strategies. The ATP‐depleted protection obtained with H3 relaxin also provides a new direction for the treatment of this particular type of inflammatory injury. The metabolic significance and inflammation‐sensing function of endogenous relaxin‐3, as well as its value for further research, are described.

## CONFLICT OF INTEREST

All the authors declared no competing interests.

## AUTHOR CONTRIBUTIONS

JL, KY and RA designed the study. JL, KY, YC and XZ refined the process. JL, YJ and YL performed most of the experiments. YJ and YL acquired consents, samples and clinical data from patients and healthy subjects. JL and KY planed and conducted bioinformatic analysis. SY, ES, SC, JZ and GJ performed some of the experiments. JL, KY and YC analysed the data. JL wrote the manuscript. RA supervised the study and the manuscript. All authors reviewed and approved the final draft.

## Supporting information

Supplementary Material

## Data Availability

Data are available upon reasonable request to the corresponding author.
