# Supplementary material for: H3 relaxin protects against calcium oxalate crystal‐induced renal inflammatory pyroptosis
Source: Cell Prolif. 2020 Sep 18;53(10):e12902. doi: 10.1111/cpr.12902 (PMC7574868; doi:10.1111/cpr.12902)
Supplement: Supplementary file 1 — Supplementary Material [file CPR-53-e12902-s001.doc]

**Supplementary Material for**

**H3 relaxin protects against calcium oxalate crystal-induced renal inflammatory pyroptosis**

Jiannan Liu^1^, Kelaier Yang^2^, Yinshan Jin^1^, Yadong Liu^1^, Yaodong Chen^3^, Xiaohui Zhang^4^, Shiliang Yu^1^, Erlin Song^1^, Song Chen^1^, Jingbo Zhang^1^, Guanhua Jing^1^, and Ruihua An^1^

Correspondence: Ruihua An, Department of Urology, the First Affiliated Hospital of Harbin Medical University, No.23 You Zheng Street, Harbin 150001, Heilongjiang, China. Phone:86-0451-85555830; E-mail: [ruihua_an@163.com](mailto:ruihua_an@163.com)

The file includes:

Figure S1. Procedure summary for the LC-MS-based TMT labeling quantitative proteomics analyses.

Figure S2. Protein sample quality verification and KEGG enrichment analysis for Proteomics analyses.

Figure S3. The anti-inflammatory effects of H3 relaxin in vivo after 7 days treatment.

Figure S4. Crystals deposition in vivo after 7 days treatment of EG and H3 relaxin.

Figure S5. The renal protective effects of H3 relaxin to 7 days treated rats.

Figure S6. Verifying in receptor protein RXFP-1 knockout.

Figure S7-9. Graphical representation corresponding the western blots.

Table S1. The siRNA sequences for the RXFP-1 genes.

**
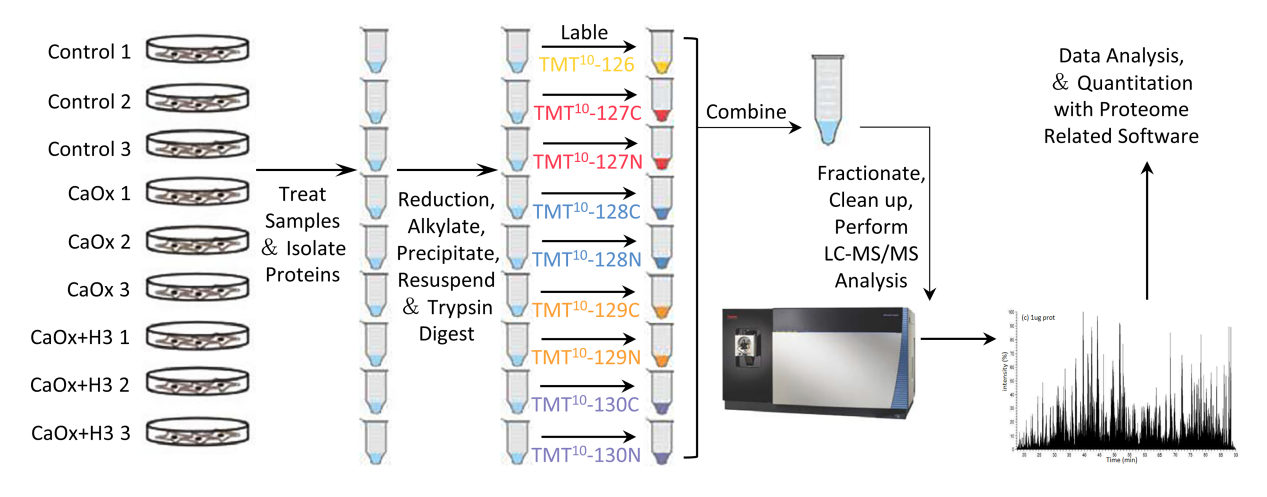
**

**Figure S1.** Procedure summary for the LC-MS-based TMT labeling quantitative proteomics analyses.

**
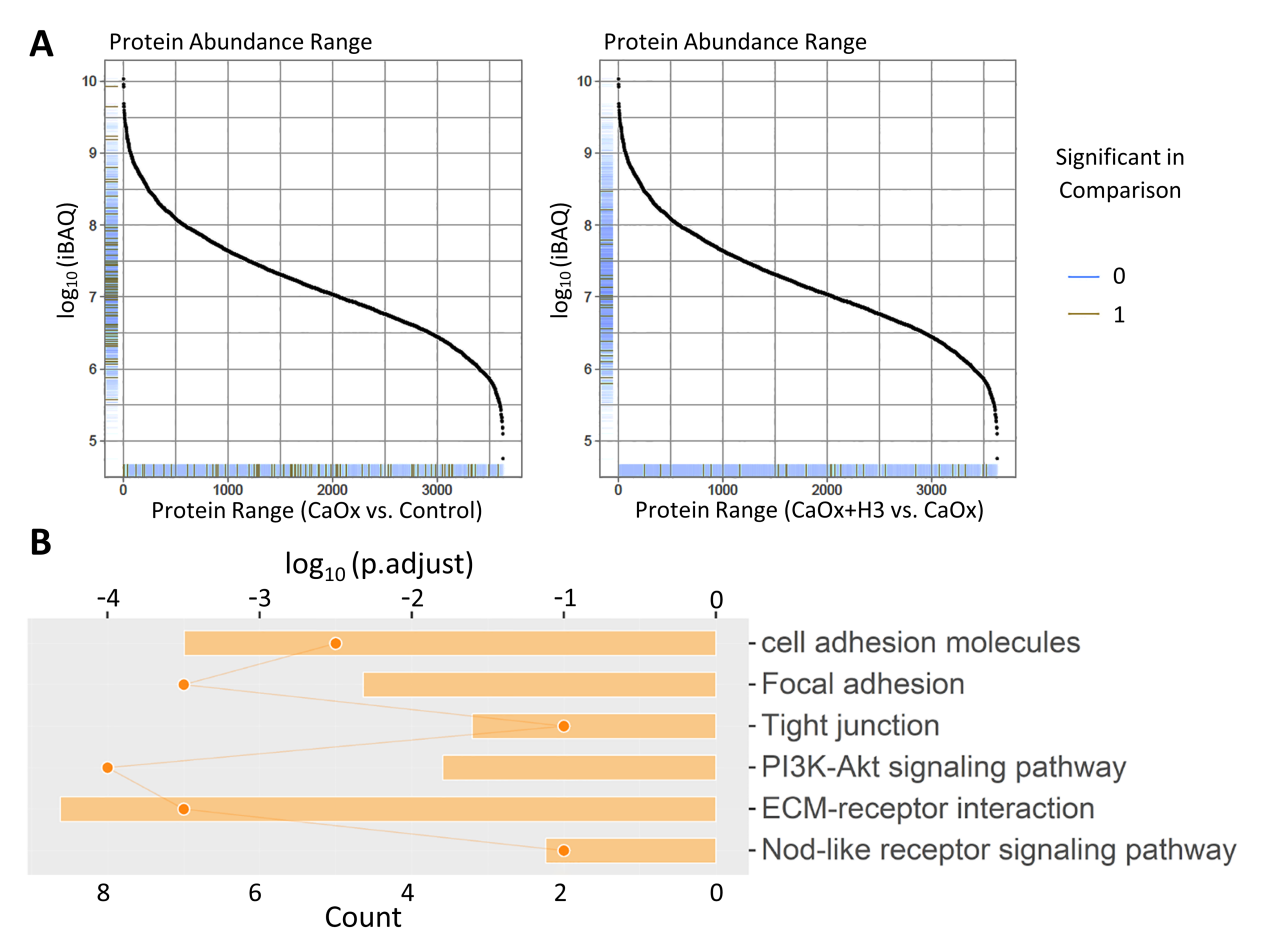
**

**Figure S2.** Protein sample quality verification and KEGG enrichment analysis for Proteomics analyses. (**A**) Protein abundance range display of CaOx group VS. control group and CaOx + H3 relaxin group vs. CaOx group, evaluated by Intensity-based absolute quantification (iBAQ). iBAQ is an approximation of protein copy numbers based on the sum of peptide-feature intensities of all peptides matching to a protein divided by the number of theoretically observable peptides. Similar trends of figures proved the homogeneity of samples. (**B**) The 104 DEPs were translated into significant biological properties using KEGG enrichment analysis. Significant pathways are shown (*P* < 0.05).


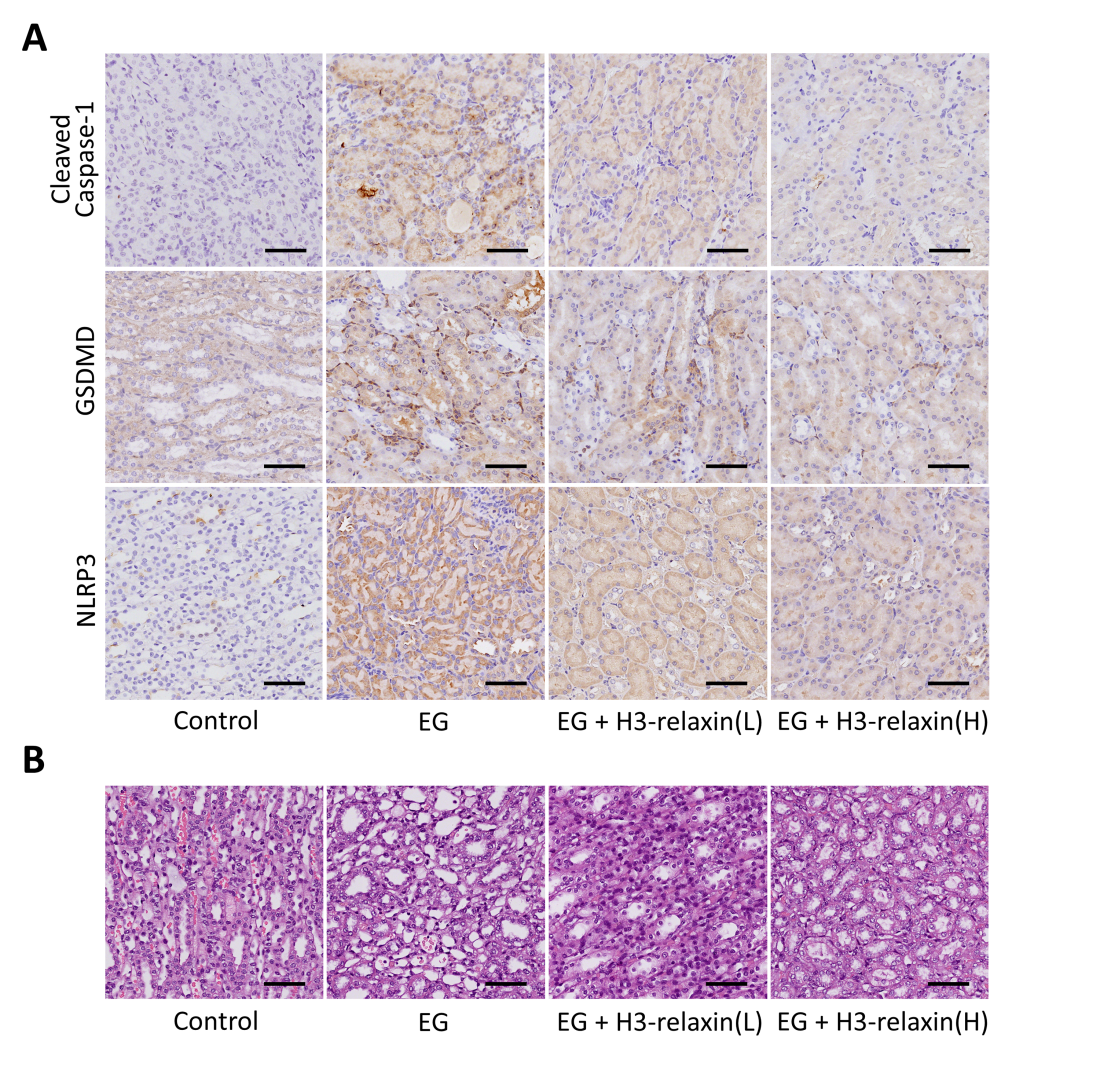


**Figure S3.** The anti-inflammatory effects of H3 relaxin in vivo after 7 days treatment. (**A**) The expression of cleaved caspase-1, cleaved GSDMD and NLRP3 were detected using Immunohistochemical assays in the treated rats kidneys (7 days), with or without H3 relaxin treated. Scale bars: 50 μm. (**B**) Hematoxylin and eosin (HE) staining of treated rats kidneys (7 days). Scale bars: 50 μm. *n* = 3 per group, representative images are shown. Although the protein expression and renal damage are less obvious than 14 days group, H3 relaxin still improved these in a dose-depend manner.


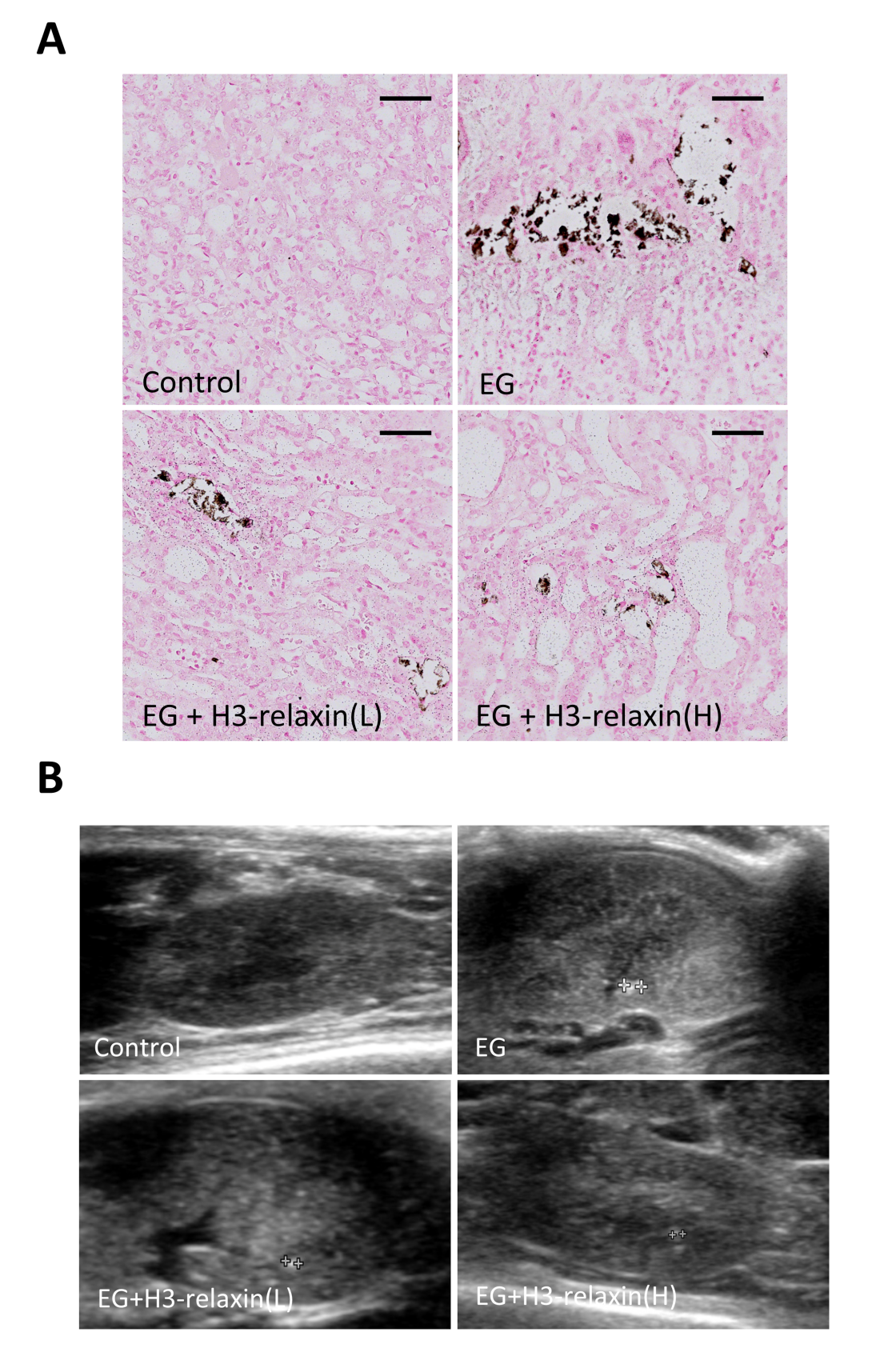


**Figure S4.** Crystals deposition in vivo after 7 days treatment of EG and H3 relaxin. (**A**) Von Kossa staining of treated rats kidneys (7 days). Scale bars: 50 μm. (**B**) Ultrasonographic examination of treated rats kidneys (7 days). *n* = 3 per group, representative images are shown.


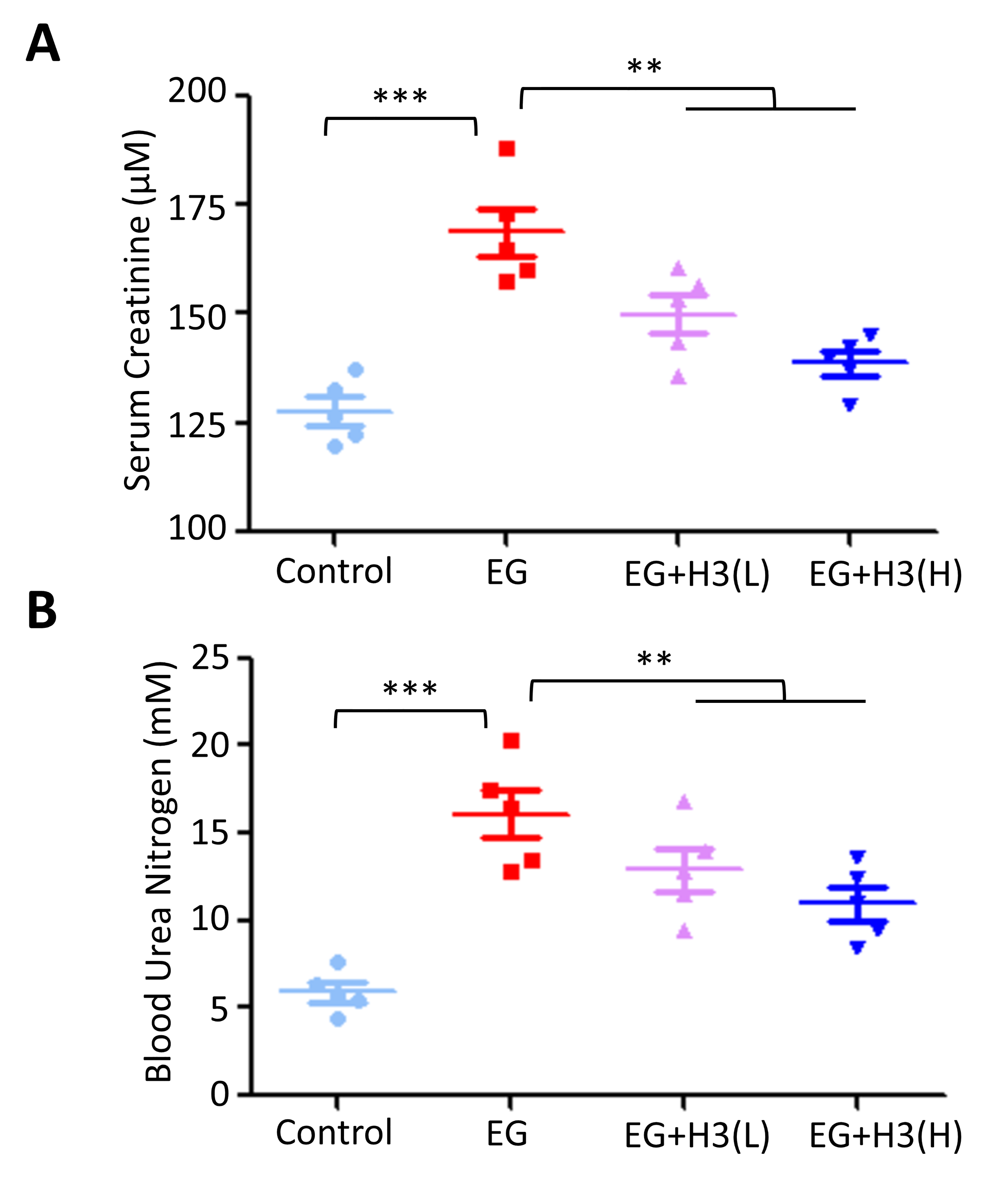


**Figure S5.** The renal protective effects of H3 relaxin to 7 days treated rats. Renal function of treated rats (7 days) was evaluated by creatinine and urea assays. *n* = 5 per group, data expressed as means ± SEM, ***P* < 0.01, ****P* < 0.001.


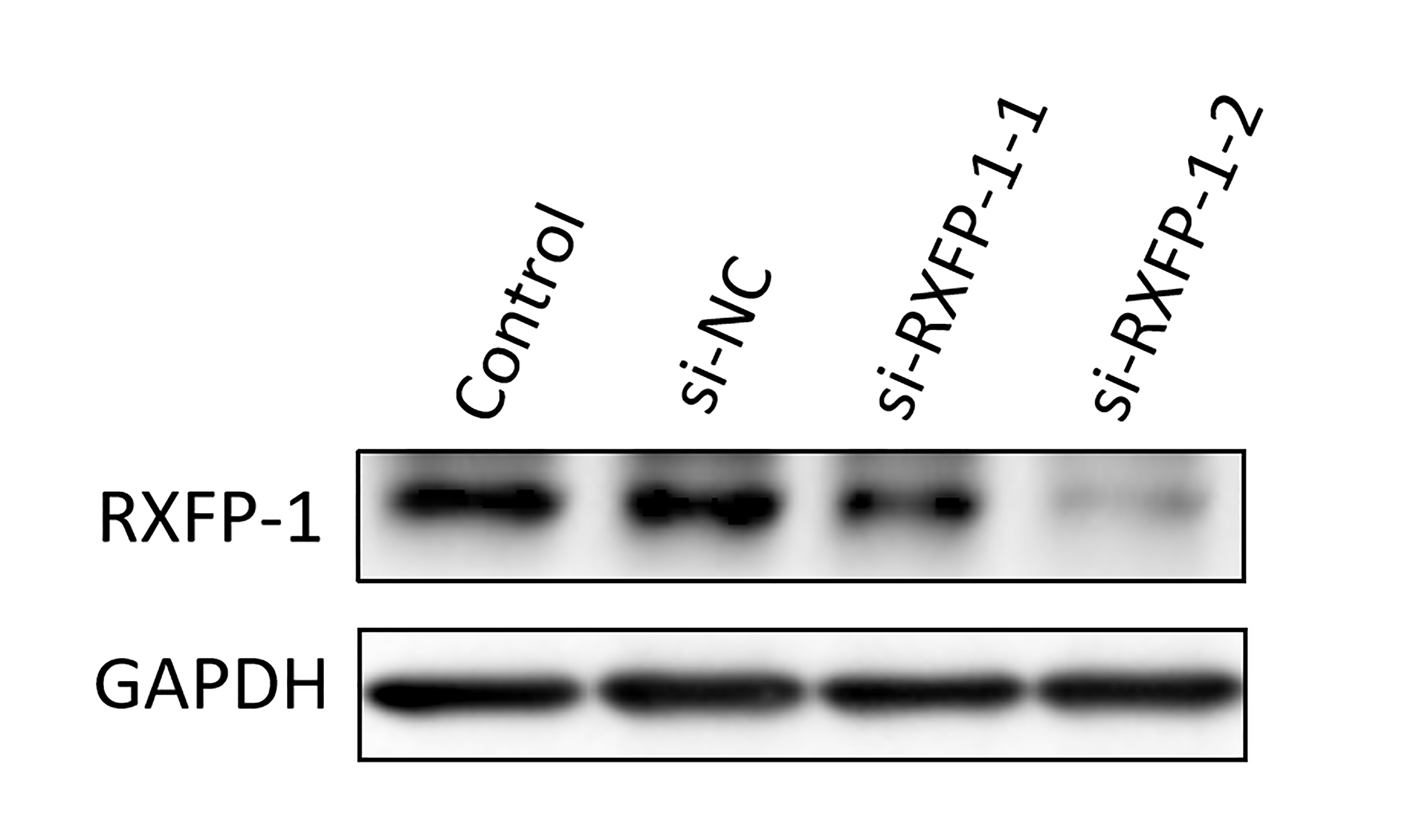


**Figure S6.** Verifying in receptor protein RXFP-1 knockout. Two different siRNA for RXFP-1 were reported to be effective. Si-RXFP-1-2 has been shown to work on TECs after repeated verification. *n* = 3 per group, representative images are shown.

**
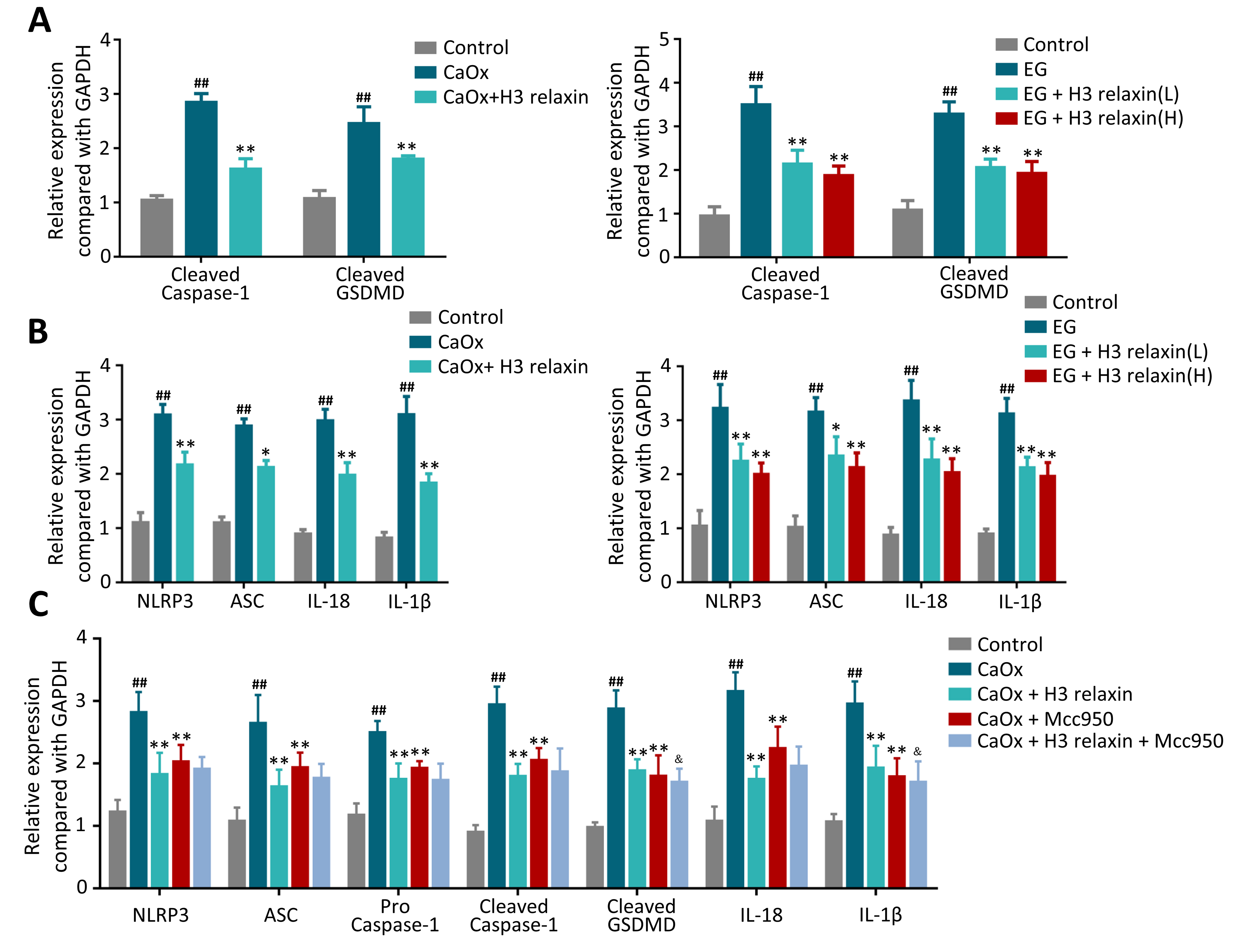
**

**Figure S7.** Graphical representation corresponding the western blots. (**A**) Graphical representation of Figure 2E. ^##^*P* < 0.01 vs. Control group, ***P* < 0.01 vs. CaOx/EG group. (**B**) Graphical representation of Figure 3A. ^##^*P* < 0.01 vs. Control group, **P* < 0.05 vs. CaOx/EG group, ***P* < 0.01 vs. CaOx/EG group. (**C**) Graphical representation of Figure 3D. ^##^*P* < 0.01 vs. Control group, ***P* < 0.01 vs. CaOx group, ^&^*P* < 0.05 vs. CaOx + H3 relaxin group. *n* = 3, data expressed as means ± SD.

**
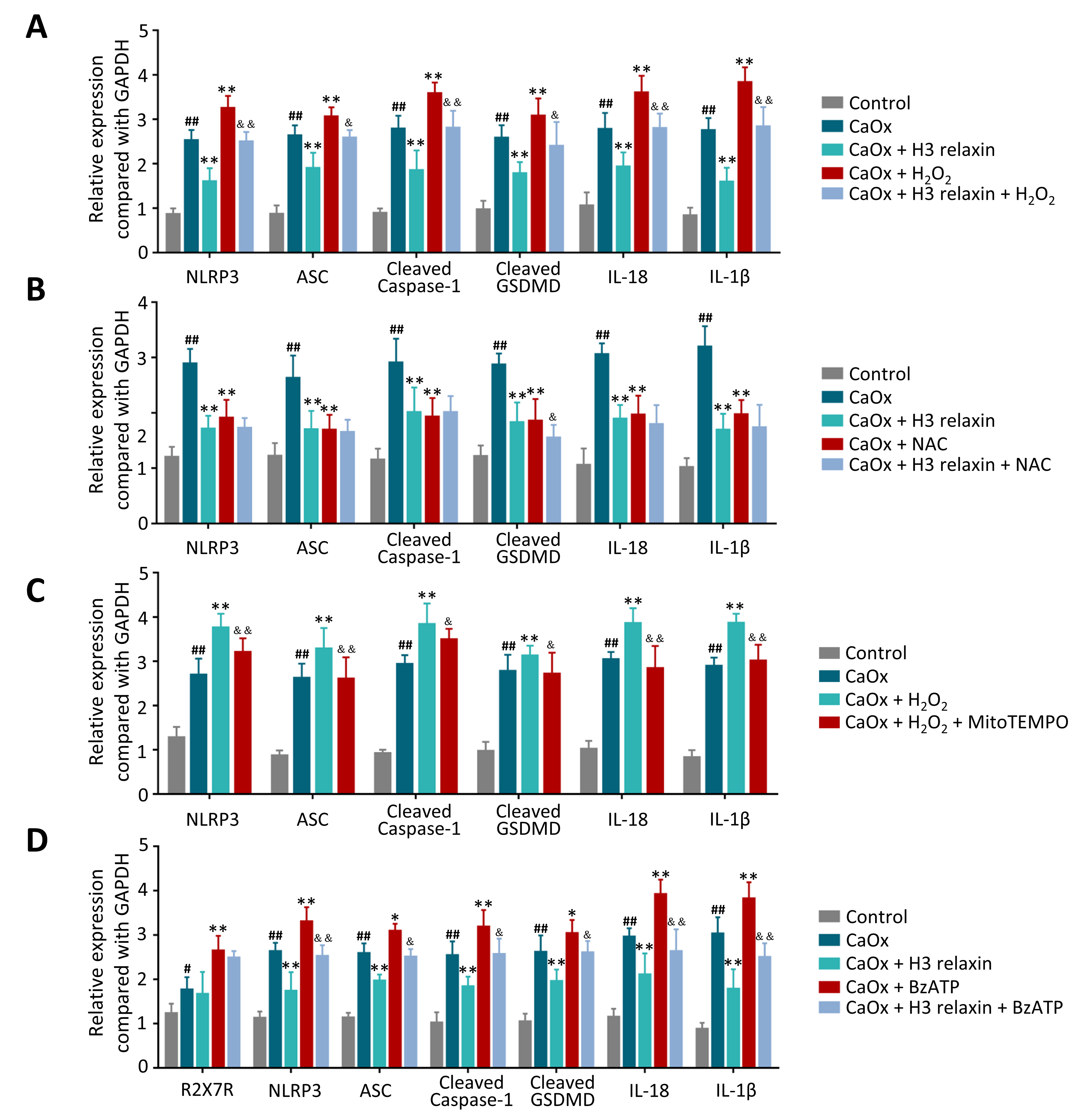
**

**Figure S8.** Graphical representation corresponding the western blots. (**A**) Graphical representation of Figure 3F. ^##^*P* < 0.01 vs. Control group, ***P* < 0.01 vs. CaOx group, ^&^*P* < 0.05 vs. CaOx + H3 relaxin group, ^&&^*P* < 0.01 vs. CaOx + H3 relaxin group. (**B**) Graphical representation of Figure 3G. ^##^*P* < 0.01 vs. Control group, ***P* < 0.01 vs. CaOx group, ^&^*P* < 0.05 vs. CaOx + H3 relaxin group. (**C**) Graphical representation of Figure 3H. ^##^*P* < 0.01 vs. Control group, ***P* < 0.01 vs. CaOx group, ^&^*P* < 0.05 vs. CaOx + H_2_O_2_ group, ^&&^*P* < 0.01 vs. CaOx + H_2_O_2_ group. (**D**) Graphical representation of Figure 4C. ^#^*P* < 0.05 vs. Control group, ^##^*P* < 0.01 vs. Control group, **P* < 0.05 vs. CaOx group, ***P* < 0.01 vs. CaOx group, ^&^*P* < 0.05 vs. CaOx + H3 relaxin group, ^&&^*P* < 0.01 vs. CaOx + H3 relaxin group. *n* = 3, data expressed as means ± SD.

**
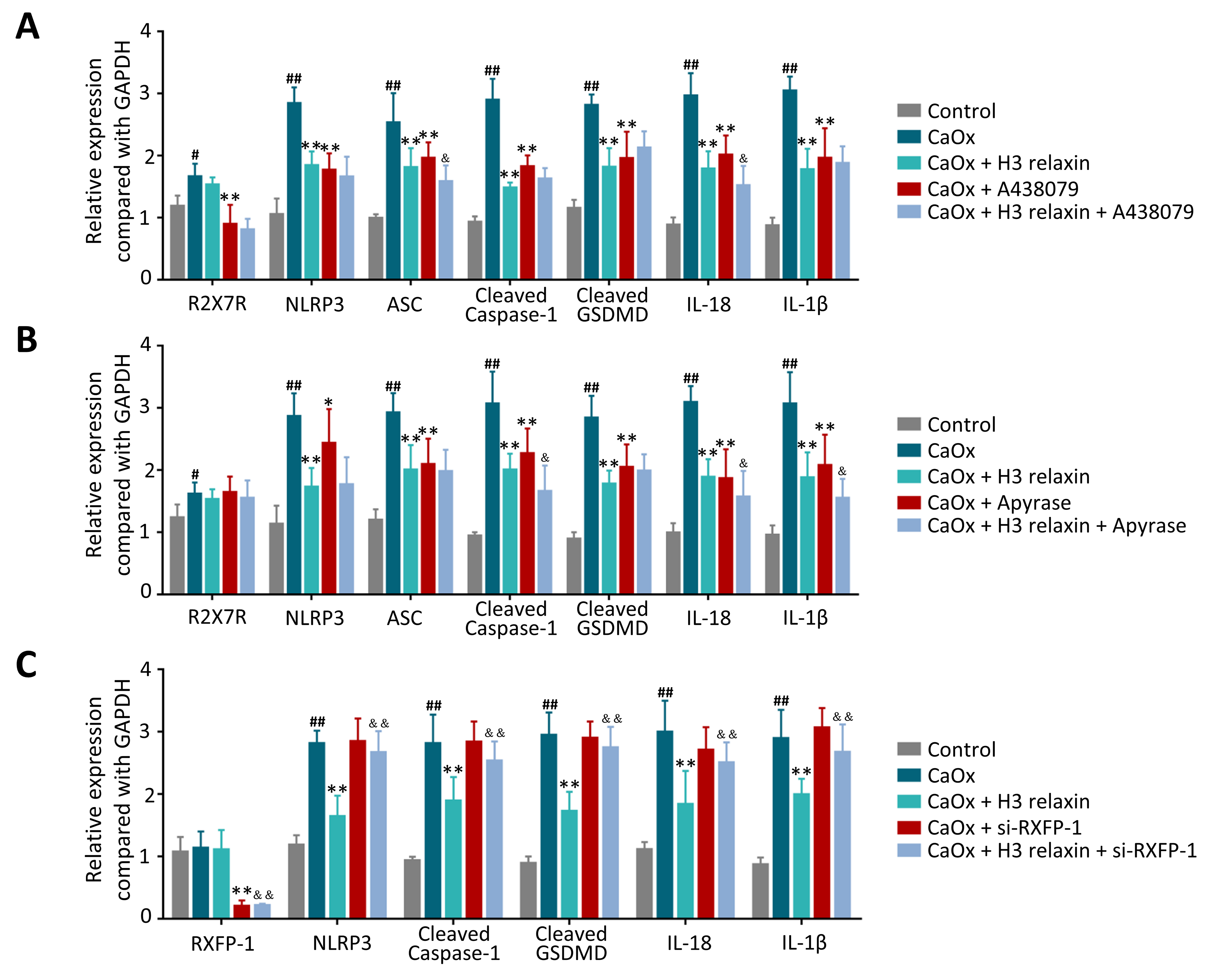
**

**Figure S9.** Graphical representation corresponding the western blots. (**A**) Graphical representation of Figure 4D. ^#^*P* < 0.05 vs. Control group, ^##^*P* < 0.01 vs. Control group, ***P* < 0.01 vs. CaOx group, ^&^*P* < 0.05 vs. CaOx + H3 relaxin group. (**B**) Graphical representation of Figure 5F. ^#^*P* < 0.05 vs. Control group, ^##^*P* < 0.01 vs. Control group, **P* < 0.05 vs. CaOx group, ***P* < 0.01 vs. CaOx group, ^&^*P* < 0.05 vs. CaOx + H3 relaxin group. (**C**) Graphical representation of Figure 6G. ^##^*P* < 0.01 vs. Control group, ***P* < 0.01 vs. CaOx group, ^&&^*P* < 0.01 vs. CaOx + si-RXFP-1 group. *n* = 3, data expressed as means ± SD.

| siRNA | Sense (5’ to 3’) |
| --- | --- |
| si-NC (random control sequence) | UUCUCCGAACGUGUCACGUTT |
| si-RXFP-1-1 | CCAUCUCCAUCUAUGCUUUTT |
| si-RXFP-1-2 | CAACUGAAAUACGGAAUCATT |

**Table S1.** The siRNA sequences for the RXFP-1 genes.
